# RNF43/ZNRF3 loss predisposes to hepatocellular-carcinoma by impairing liver regeneration and altering the liver lipid metabolic ground-state

**DOI:** 10.1038/s41467-021-27923-z

**Published:** 2022-01-17

**Authors:** Germán Belenguer, Gianmarco Mastrogiovanni, Clare Pacini, Zoe Hall, Anna M. Dowbaj, Robert Arnes-Benito, Aleksandra Sljukic, Nicole Prior, Sofia Kakava, Charles R. Bradshaw, Susan Davies, Michele Vacca, Kourosh Saeb-Parsy, Bon-Kyoung Koo, Meritxell Huch

**Affiliations:** 1 The Max Planck Institute of Molecular Cell Biology and Genetics, 01307 Dresden, Germany; 2 The Wellcome Trust/CRUK Gurdon Institute, University of Cambridge, Cambridge, CB2 1QN UK; 3 Wellcome Trust - Medical Research Council Stem Cell Institute, University of Cambridge, Gleeson Building, Cambridge, CB2 1QR UK; 4 Department of Genetics, University of Cambridge, Cambridge, CB2 3EH UK; 5 Wellcome Sanger Institute, Wellcome Genome Campus, Hinxton, Cambridge, CB10 1SA UK; 6 Biomolecular Medicine, Division of Systems Medicine, Department of Metabolism, Digestion and Reproduction, Imperial College London, London, SW7 2AZ UK; 7 Department of Histopathology, Cambridge University Hospitals NHS Foundation Trust, Cambridge, UK; 8 Metabolic Research Laboratories, University of Cambridge, Cambridge, UK; 9 Clinica Medica Cesare Frugoni, Department of Interdisciplinary Medicine, University of Bari “Aldo Moro”, Bari, Italy; 10 Department of Surgery, University of Cambridge and NIHR Cambridge Biomedical Research Centre, Cambridge, UK; 11 Institute of Molecular Biotechnology of the Austrian Academy of Sciences (IMBA), Vienna Biocenter (VBC), Dr. Bohr-Gasse 3, 1030 Vienna, Austria

**Keywords:** Hepatocellular carcinoma, Cancer models

## Abstract

RNF43/ZNRF3 negatively regulate WNT signalling. Both genes are mutated in several types of cancers, however, their contribution to liver disease is unknown. Here we describe that hepatocyte-specific loss of *Rnf43*/*Znrf3* results in steatohepatitis and in increase in unsaturated lipids, in the absence of dietary fat supplementation. Upon injury, *Rnf43*/*Znrf3* deletion results in defective hepatocyte regeneration and liver cancer, caused by an imbalance between differentiation/proliferation. Using hepatocyte-, hepatoblast- and ductal cell-derived organoids we demonstrate that the differentiation defects and lipid alterations are, in part, cell-autonomous. Interestingly, ZNRF3 mutant liver cancer patients present poorer prognosis, altered hepatic lipid metabolism and steatohepatitis/NASH signatures. Our results imply that RNF43/ZNRF3 predispose to liver cancer by controlling the proliferative/differentiation and lipid metabolic state of hepatocytes. Both mechanisms combined facilitate the progression towards malignancy. Our findings might aid on the management of those RNF43/ZNRF3 mutated individuals at risk of developing fatty liver and/or liver cancer.

## Abstract (web summary)

The E3 ubiquitin ligases RNF43/ZNRF3 are often mutated in cancer but their precise contribution to liver disease is unknown. Here, the authors show that RNF43/ZNRF3 alterations predispose to liver cancer by controlling the differentiation and lipid metabolic state of hepatocytes.

## Introduction

WNT signalling regulates many cellular processes^1^. The key switch in the canonical Wnt pathway is β-catenin. In the absence of WNT signal, β-catenin is targeted for degradation^2^, whereas upon WNT ligand binding to FZDs and LRP5/6, β-catenin accumulates in the cytoplasm and nucleus, where it engages its effectors, the DNA-bound T cell factor (TCF) transcription factors^3,4^. In the liver, WNT signalling plays a critical role in tissue regeneration^5–8^ and metabolic zonation^9,10^, as well as in the establishment and progression of liver diseases, including liver cancer. For instance, ablation of some components of the pathway (β-catenin, the LGR4/5, TCF4, APC), results in impaired activation and completion of the regenerative process^11–14^ and altered liver zonation^11–16^.

*Rnf43* and *Znrf3* are two negative-feedback regulators of the WNT pathway^17,18^. Both are E3-ubiquitin ligases that specifically ubiquitinate the cytoplasmic loops of the FZDs receptors, which induces their rapid endo-lysosomal degradation^17,18^. Conversely, R-spondins (RSPO), ligands of LGR4-5-6 receptors, also interact with RNF43/ZNRF3, which reverses the RNF43/ZNRF3-mediated membrane clearance of FZDs boosting WNT signal strength and duration^19^. Mutations in *RNF43* and/or *ZNRF3* have been observed in human cancers^20^. We had reported that intestinal deletion results in adenoma formation^18^. In primary liver cancer, *ZNRF3* is mutated in hepatocellular carcinoma (HCC)^21^, while *RNF43* in intrahepatic cholangiocarcinoma^22^. However, their role in liver disease has not been explored yet. Ubiquitous (whole-body) deletion of both *Rnf43/Znrf3* results in some liver metabolic changes^12^; however, whether these genes exert a direct regulation on liver metabolism or whether these result from secondary effects on non-liver tissues has not been elucidated yet.

Here, we describe that specific deletion of RNF43/ZNRF3 in adult hepatocytes results in liver degeneration, steatohepatitis, increased unsaturated lipids and altered liver lipid zonation. Concomitantly to these alterations, *Rnf43*/*Znrf3* loss increases hepatocyte proliferation. Both alterations are explained, at least in part, by cell-autonomous mechanisms, since *Rnf43*/*Znrf3-null* liver epithelial organoids exhibit significant intracellular lipid droplet accumulation and reduced differentiation capacity. Upon chronic injury, the lipotoxicity and imbalance in proliferation/differentiation states contribute to a defective regeneration, extensive fibrosis and tissue damage, which, with time, progresses to malignancy, either HCC or mixed HCC/CC cancer. Notably, our mutant mouse recapitulates the signatures of HCC patients with mutations in *RNF43* and/or *ZNRF3*, which also present lipid metabolic alterations and poorer prognosis. Our observations imply that RNF43/ZNRF3 predispose to liver cancer, at least in part, by altering the hepatocyte metabolic ground-state while, in parallel, preventing the tissue from completely regenerating upon damage. Our results are in agreement with our previously published preprint manuscript (Mastrogiovanni et al.^23^) and the recent publication by Sun et al., published while our manuscript was under revision^24^.

## Results

### *Rnf43/Znrf3* loss results in hyperplasia and tissue degeneration

**Fig. 1:**
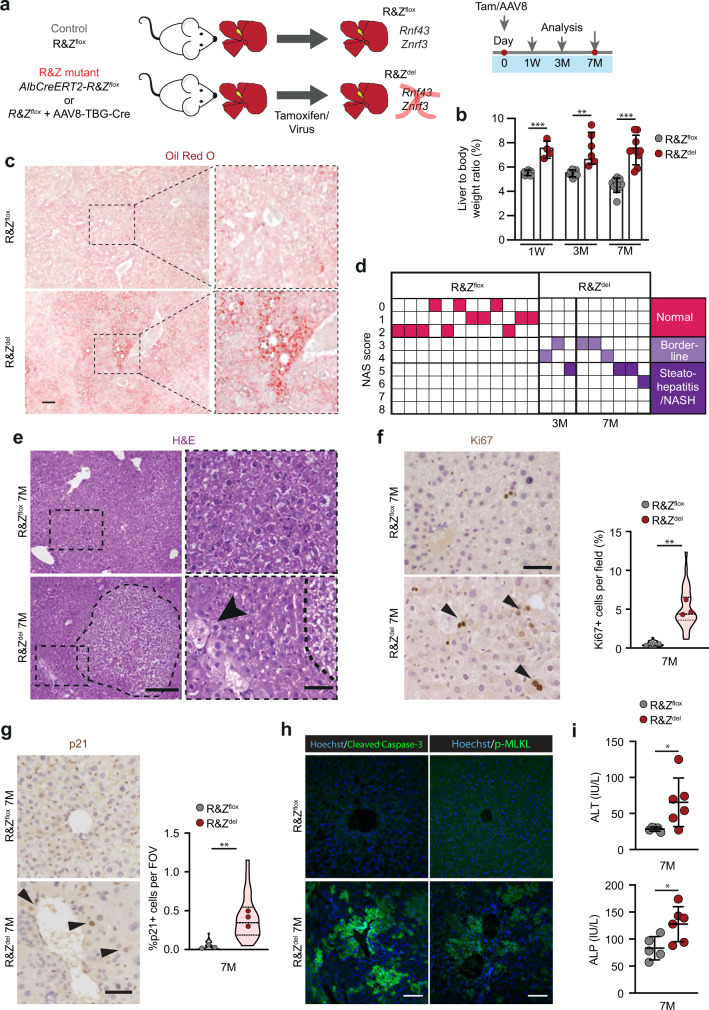
*Rnf43/Znrf3* (R&Z) deletion induces hepatocyte proliferation, hepatomegaly, tissue degeneration and steatohepatitis. **a** Experimental design. Liver-specific *Rnf43/Znrf3*^*del*^ mice (R&Z^del^) were generated by injecting AAV8-TGB-Cre virus to 8–12week-old *Rnf43/Znrf3*^*flox*^ mice (R&Z^flox^) or by injecting tamoxifen (Tam) to 8–12-week-old *AlbCre-ERT2-R&Z*^*flox*^ mice. Control R&Z^flox^ mice received AAV8-null virus or vehicle. Livers were collected at 1 week (1W), 3 months (3 M) or 7 months (7M) later. Red dot, blood collection. **b** R&Z^del^ results in hepatomegaly. The graph represents the percentage of liver-to-body weight ratio. Results are presented as mean + /− SD from R&Z^flox ^1W, *n* = 5; R&Z^del ^1W; R&Z^flox ^3M, *n* = 7; R&Z^del ^3M, *n* = 6; R&Z^del ^7M, *n* = 9; R&Z^flox ^7M, *n* = 10. Unpaired two-tail *t* test. 1W, ***p = 0.0007; 3M, **p = 0.0017; 7M, ***p = 0.0001. **c** Oil red-O staining in R&Z^del^ 3M. Representative pictures of *n* = 3 independent experiments. Scale bar, 100 μm. **d** NAS scoring. Each column represents an independent mouse. Pink = NAS score 0–2 = Normal; clear violet = NAS score 3–4= borderline; dark violet = NAS score 5–8= steatohepatitis. **e** Representative histopathological analysis showing nodules (dashed circle) with cellular degeneration and ballooned hepatocytes (arrowheads) in R&Z^del^ livers (*n* = 7). Scale bar, 100 μm (left) and 50 μm (right). **f** R&Z^del^ livers present a significant increase in the number of proliferating (Ki67+, arrowheads) cells. Representative pictures. Scale bar, 50 μm. Graphs represent the percentage of Ki67+ hepatocytes per field-of-view (FOV). Violin plot represents median, IQR and full distribution of all FOVs (*n*  = 10 per mouse). Dots, mean of all FOV per mouse (*n*  = 3). Unpaired two-tail *t* test of means, **p = 0.0011. **g** Representative pictures of liver sections stained for p21 (arrowheads, p21+ cells). Scale bar, 50 μm. The graph represents the quantification of p21+ cells at 7M of age. Data are represented as violin plots showing median, IQR and full distribution of FOVs (*n*  = 10). Dots, mean of all FOV per mouse (*n*  = 3). Unpaired two-tail *t* test of means, **p = 0.0034. **h** Cleaved Caspase-3 and p-MLKL staining in control (top) and R&Z^del^ (bottom). Representative images from *n* = 3 independent 7M old mice. Scale bar, 100 μm. **i** Serum levels for ALT and ALP. Data represent mean + /− SD of *n* = 5 R&Z^flox^ and *n* = 6 R&Z^del^ samples. Unpaired two-tail *t* test. ALT, *p = 0.0376; ALP, *p = 0.0279. Source data are provided as a Source data file.

To study the liver-specific role of *Rnf43/Znrf3*, we took advantage of our *Rnf43/Znrf3*^*flox*^ conditional alleles^18^, which allow specific deletion of the two genes simultaneously. *Rnf43* and *Znrf3* were expressed in hepatocytes (Supplementary Fig. 1a), in agreement with ref. ^12^, but also in EpCAM^+^ ductal cells and CD11b^+^ macrophages (*Znrf3* only) (Supplementary Fig. 1a). Therefore, to generate hepatocyte-specific *Rnf43/Znrf3* mutant mice—*Rnf43/Znrf3*^*del*^ mice, from here on—we either injected *Rnf43/Znrf3*^*flox*^ mice with a hepatotropic AAV8-TGB-Cre virus or crossed *Rnf43/Znrf3*^*flox*^ mice with a tamoxifen-inducible *AlbCre-ERT2* reporter^25^, to generate the compound mice *AlbCre-ERT2/Rnf43-Znrf3*^*flox*^. Recombination was induced in adult mice (8 weeks or older) (Fig. 1a) and deletion was confirmed for all time points analysed (Supplementary Fig. 1b). No recombination was detected in non-induced mice (*Rnf43/Znrf3*^*flox*^, from hereon) (Supplementary Fig. 1c).

Following recombination, we collected *Rnf43/Znrf3*^*del*^ and control livers at 1 week and 3 months after deletion (Fig. 1a). *Rnf43/Znrf3*^*del*^ mice exhibited significant hyperplasia and hepatomegaly at 1 week (Fig. 1b), which persisted throughout the course of the study, resembling the hepatocyte-specific *Apc* mutant mice^16^. This correlated with a significant increase in the number of proliferating hepatocytes (Ki67^+^) (Supplementary Fig. 1d, e). At the histological level, we noted that at 3 months, but not at 1 week, double *Rnf43/Znrf3*^*del*^ mutant livers presented signs of cellular degeneration (hepatocyte ballooning, decellularization) (Supplementary Fig. 1d, haematoxylin and eosin (H&E) panel arrowhead), which coincided with a significant increase in the number of apoptotic (Cleaved Caspase-3+) and necroptotic (p-MLKL+) positive hepatocytes (Supplementary Fig. 1f). In addition, we observed a significant increase in innate (NK and macrophages) as well as adaptative (T cells, CD3+) inflammatory cells (Supplementary Fig. 1g). Notably, the mutant livers presented foci of fat deposition, as assessed by Oil Red-O staining (Fig. 1c). Tissue degeneration, inflammation, fat deposition and ballooned hepatocytes are observed in a variety of acute and chronic liver diseases^26^, including steatohepatitis/nonalcoholic steatohepatitis (NASH; inflamed fatty liver)^26–28^. Hence, we formally determined the levels of steatohepatitis in our mice by obtaining the non-alcoholic fatty liver disease (NAFLD) Activity Score (NAS score, Supplementary Fig. 2a)^29^. NAS score indicated that double mutant mice presented a probable/borderline score, with 1 mouse scoring >5 (steatohepatitis) (Fig. 1d and Source data). Individual homozygous mutants and wild-type (WT) littermate controls did not present any signs of hepatomegaly, neither discernible histological architecture changes (inflammatory foci, fat accumulation, tissue degeneration) nor steatohepatitis (NAS score <2) (Supplementary Fig. 1h–j). Hence, we opted to not study the single mutants any further.

The significant increase in the number of proliferating hepatocytes, combined with tissue damage features (inflammation and cell death) and probable steatohepatitis at 3 months post deletion, prompted us to investigate the impact of long-term depletion of both genes. We opted to investigate a maximum of 7 months post-deletion, since this represented a total mouse age of a maximum of 10 months and prevents the occurrence of confounding factors due to ageing, and the well-documented aged-induced senescence changes that arise in middle-aged and old-aged mice^30^. At this later time point, *Rnf43/Znrf3*^*del*^ livers presented exacerbated hepatocyte proliferation (Ki67) and cellular degeneration with clear cell nodules formed by hepatocytes with nuclear alterations (crenation and karyorrhexis) and ballooned, and a significant number of apoptotic (Cleaved Caspase-3+), necroptotic (p-MLKL+) and senescent (P21+) hepatocytes, in agreement with the significant elevation of the liver serum parameters aspartate transaminase (AST) and alkaline phosphatase (ALP) (Fig. 1e–i). In addition, 50% of the mice exhibited steatohepatitis (NAS score >5) while the remainder scored probable/borderline (NAS score 3–4) (Fig. 1d and Supplementary Fig. 2a). Of note, NASH in humans refers to a disease that develops in the context of obesity and systemic dysmetabolic alterations. However, none of the mice exhibited any of these features. Instead, body weight, triglycerides (TG) and cholesterol levels were under the normal range at 7 months post deletion (Supplementary Table 1). Remarkably, at this later time point, 10% of the mice (2 out of 20) had developed adenomas (Supplementary Fig. 2b), suggesting progression towards malignancy. As expected, mutant livers also presented a marked increase in the Wnt/β-catenin liver zonated genes (GS and CYP2E1) (Supplementary Fig. 2c, d) and higher levels of β-CATENIN around the central vein area (Supplementary Fig. 2e). None of these histological changes was detected in any of the control WT littermates (non-tamoxifen or control-virus treated) (Supplementary Fig. 2f).

### RNF43/ZNRF3 control liver lipid metabolic gene expression

**Fig. 2:**
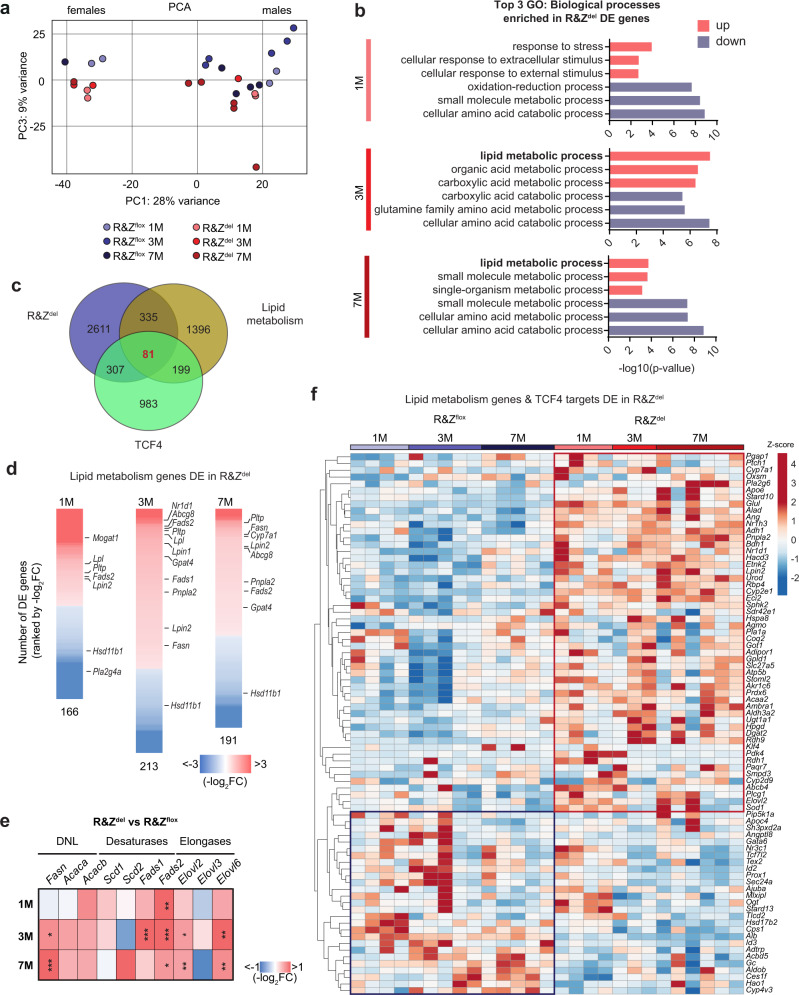
*Rnf43* and *Znrf3* (R&Z) null hepatocytes present lipid metabolic transcriptional changes. **a**–**f** Liver tissues from 1- 3- and 7-month-old R&Z^del^ mice and R&Z^flox^ littermates were collected and processed for RNAseq analysis. Differentially expressed (DE) gene profiles were obtained as described in “Methods.” **a** Principal component analysis (PCA) of R&Z^flox^ and R&Z^del^. Each data point represents one sample. Note that PC1 is strongly correlated with gender, whereas PC3 separates R&Z^flox^ mice from R&Z^del^ mice. PC2 corresponded to the two batches (batch1 and batch 2) from which the data were generated. **b** Graphs showing top 3 gene ontology (GO) terms significantly enriched for genes upregulated (red) and downregulated (purple) in R&Z^del^ compared to R&Z^flox^. Full list in Supplementary Data 1. **c** Venn diagram showing a correlation between genes involved in lipid metabolism (brown), TCF4 target genes (green) and genes DE at any time point in R&Z^del^ livers (purple). The numbers denote the number of genes in each comparison. Details are given in Supplementary Data 3. **d**, **e** Heatmaps of the lipid metabolism genes DE in R&Z^del^ livers. **d** Genes DE averaged between all mice per time point and ranked by fold change with respect to their respective R&Z^flox^ control. **e** DE genes showing some representative lipid metabolic genes. Likelihood ratio test and Benjamini–Hochberg correction; *Fasn*, 3M *p = 0.0446, 7M ***p = 0.0001; *Fads1*, 3M ***p = 0.0006; *Fads2*, 1M ***p = 0.0038, 3M ***p = 0.0001, 7M *p = 0.0282; *Elovl2*, 3M *p = 0.0392, 7M **p = 0.0088; *Elovl6*, 3M **p = 0.0020, 7M **p = 0.0088. **f** Heatmap of the RPKM values (raw *z*-scored) of DE lipid metabolic genes found to be TCF4 targets. Each column, one independent biological replicate. Source data are provided as a Source data file.

We next performed global gene expression analysis (RNA sequencing (RNAseq)) of *Rnf43/Znrf3*^*del*^ and corresponding controls (*Rnf43/Znrf3*^*flox*^) at several time points after gene deletion (1, 3 and 7 months). Principal component analysis (PCA) revealed that *Rnf43/Znrf3*^*del*^ mutants clustered together and separated from control littermates in both female and male mice (Fig. 2a). Gene ontology (GO) and Ingenuity Pathway Analysis (IPA) of the differentially expressed (DE) genes in *Rnf43/Znrf3*^*del*^ (compared to controls) indicated that the majority of the upregulated genes at 1 month after deletion correspond to inflammation, cell damage, oxidation and responses to stress and external stimulus, in agreement with our histopathological analysis (Fig. 2b, Supplementary Fig. 3a and Supplementary Data 1 and 2). Wnt signalling and its related effectors were within the top potential upstream regulators at the three time points (Supplementary Fig. 3b and Supplementary Data 2). Of note, pericentral and periportal genes were upregulated and downregulated, respectively, compared to WT mice (Supplementary Fig. 3c), suggesting that liver zonation was inverted and confirming the reported role of canonical WNT signalling in the maintenance of liver zonation^12,15^. We then overlapped our DE gene list with a published list of liver TCF4 targets obtained by chromatin immunoprecipitation–sequencing^15^. We found that 11.6% of our DE genes (388 of 3334 genes) were canonical liver WNT/TCF4 targets, such as *Axin2*, *Sp5*, *Cyp2e1* and *Tnfrsf19*, among others (Fig. 2c, Supplementary Fig. 3d and Supplementary Data 3_S3 and S9). More interestingly, both IPA and GO analysis revealed that a significant proportion of the upregulated genes were involved in lipid/phospholipid metabolism, being “lipid metabolic process” the topmost significantly enriched category at 3-months post-deletion (Fig. 2b, Supplementary Fig. 3a and Supplementary Data 1 and 2).

To further investigate the lipid metabolic changes observed, we generated a gene list of lipid metabolic genes (see methods) and overlapped it to our DE gene lists (3334 genes). We found that ~12.5% of all the DE genes (416 out of 3334) were involved in lipid metabolic processes (Fig. 2c). Within these, we found genes encoding for enzymes involved in the synthesis and remodelling of fatty acids including *Fasn* (fatty acid synthase), *Fads1* and *Fads2*, which regulate unsaturation of fatty acids through the introduction of double bonds on the fatty acyl chain and elongases, like *Elovl2*, *Elovl3* and *Elovl6*, which add 2 carbons to the long- and very-long-chain fatty acids. In addition, we found significantly upregulated genes involved in diacylglycerol (DAG) biosynthesis (i.e. *Mogat1*, *Lpin2* and *Lpin1)* and in the biosynthesis of TG and phospholipids, such as *Dgat2* (synthesis of TG from DAG) and *Etnk2* (which catalysers the first step of phosphatidylethanolamine (PE) biosynthesis). Also, genes involved in lipid transfer (*Pltp* and *Lpl*) and TG hydrolysis (*Pnpla2*, which generates DAG from TG) were also significantly upregulated. Last, we found upregulation in the expression of genes involved in cholesterol and bile acid metabolism, such as the oxysterol sensor Liver X receptor alpha (*Nr1h3*) and its target genes *Cyp7a1* (the rate-limiting step enzyme in cholesterol conversion into bile acids), *Abcg8* (cholesterol secretion in bile), as well as the phospholipid transporter *Abcb4* (i.e. MDR2). Conversely, genes involved in phospholipid oxidation (*Pla2g4a*) and cholesterol hydroxylation (*Hsd11b1*) were within the most downregulated genes (Fig. 2d, e, Supplementary Fig. 3e and Supplementary Data 3).

We next compared the genes DE to the liver TCF4 targets^15^ and overlapped it to the list of lipid metabolic genes. We found that 19.5% of all DE genes catalogued as lipid metabolic genes were also bona fide TCF4/canonical WNT targets (81 out of 416, Fig. 2c) including *Nr1h3, Cyp7a1* (cholesterol) and phospholipid, DAG, TG and FA biosynthesis genes such as *Etnk2*, *Lpin2*, *Dgat2* and *Elvol2* and the phospholipid remodelling gene *Pla2g6*, among others (Fig. 2f and Supplementary Data 3). These results suggest that RNF43/ZNRF3 regulate lipid metabolic gene expression through both the activation of canonical WNT signalling as well as non-WNT related mechanisms.

### RNF43/ZNRF3 as gatekeepers of the liver lipid metabolic ground state

**Fig. 3:**
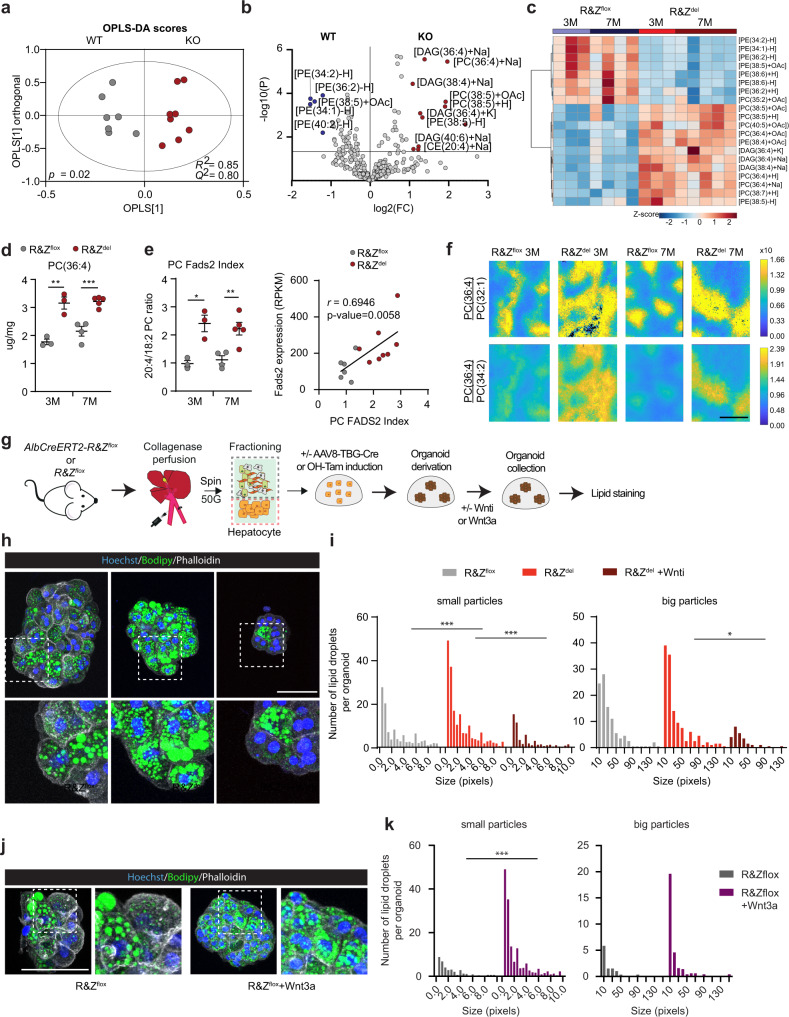
*Rnf43*/*Znrf3*^*del*^ livers present altered lipid composition and zonation and cell-autonomous accumulation of lipid droplets. **a**–**e** Lipidomics analysis in the mutant (R&Z^del^) and control (R&Z^flox^) mice at 3 (3M) and 7 months (7M) post-deletion. **a** Orthogonal Partial Least Squares Discriminant Analysis (OPLS-DA) model of hepatic lipid profiles discriminates between genotypes. *R*^2^ = 0.85, *Q*^2^ = 0.80, *p* = 0.02. **b** Volcano plot highlights significantly changed lipids; horizontal line shows *p* = 0.05 as determined by unpaired *t* test. **c** Heatmap analysis of lipid features across the two genotypes and time points. The top 20 features (based on ANOVA) are shown. **d** PC(36:4) abundance. Unpaired two-tail *t* test. 3M, **p = 0.0042; 7M, ***p = 0.0005. **e** PC Fads2 index and RNA correlation. Left, Fads2 index [PC(36:4) + PC(38:4)/PC(34:2) + PC(36:2)]. Data are represented as mean + /− SD of *n* = 3 (3M) and *n* = 4–5 (7M) mice per group. Unpaired two-tail *t* test. 3M, *p = 0.0103; 7M, **p = 0.0059. Right, the correlation between PC Fads2 index and *Fads2* RNA expression. Pearson correlation, *r* = 0.69, CI 95% (0.2597–0.8952), two-tailed *p* = 0.005. **f** Mass spectrometric imaging analysis shows increased intensity and expansion of area covered by PC(36:4) relative to PC(32:1) and PC(34:2) in R&Z^del^ liver. **g**–**k** Isolated adult hepatocytes derived from either *Alb*CreERT-R&Z^flox^ or R&Z^flox^ livers were expanded and treated or not with hydroxy-tamoxifen (OH-Tam) or AAV8-TGB-Cre infection to induce *Rnf43/Znrf3* deletion in vitro. The derived organoid lines and parental non-recombined lines were treated with Wnt inhibitors (Wnti, **h**, **i**) or WNT3A conditioned media (Wnt3a, **j, k**) and analysed for lipid droplet content by Bodipy staining. **g** Experimental design. **h**–**k** Bodipy staining and quantification of R&Z^flox^ and R&Z^del^ hepatocyte organoids grown in control medium or medium supplemented with Wnti. **h** Representative pictures from *n* = 2 independent experiments. Scale bar, 100 μm. **i** Image analysis quantification of the intracellular lipid droplets size and content (*n*  = 5). Two-way ANOVA with Tukey’s multiple comparisons. Small particles, R&Z^flox^vsR&Z^del^ ***p = 0.0001; R&Z^del^vsR&Z^del^ + Wnti ***p = 0.0001. Big particles, R&Z^del^vsR&Z^del^ + Wnti *p = 0.0198. **j**, **k** Bodipy staining and quantification of R&Z^flox^ *wild-type* hepatocyte organoids grown in control or WNT3A media. **j** Representative pictures from *n* = 2 independent experiments. Scale bar, 100 μm. **k** Image analysis quantification of the intracellular lipid droplets size and content (*n*  = 5), two-way ANOVA, *p = 0.0000. Source data provided in Source data file.

To determine whether the gene expression changes translated into liver lipid alterations, we performed untargeted lipidomics on the *Rnf43/Znrf3*^*del*^ and control *Rnf43/Znrf3*^*fox*^ livers at 3 and 7 months post deletion. We detected several hundred lipid species, including TG, phosphatidylcholines (PC), PE, free fatty acids, ceramides, sphingomyelins, DAGs, phosphatidylinositols (PI), cardiolipins, and lysophosphatidylcholines (LPC) (Supplementary Data 4). The analysis of the individual lipid species separated *Rnf43/Znrf3*^*del*^ from the *Rnf43/Znrf3*^*flox*^ mice (Fig. 3a). A direct comparison of the lipid profiles revealed that mutant *Rnf43/Znrf3*^*del*^ livers presented an increase in phospholipids and DAGs containing 4 or 5 double bonds (e.g. DAG(36:4), DAG(38:4), PC(36:4), PE(38:4), PC(38:5)), compared to WT littermate controls. In contrast, phospholipids with 1 or 2 double bonds (e.g. PE(34:1), PE(34:2), PE(36:2), PC(35:2)) were significantly reduced (Fig. 3b–d and Supplementary Data 4). These results suggested the remodelling of the fatty acyl chain composition of phospholipids and diacylglycerides and were in agreement with the gene expression signatures (Fig. 2), that had shown a significant upregulation of the genes involved in TG, DAG and PC and PE biosynthesis and elongases (e.g. *Dgat2*, *Etnk2*, *Fads1* and *Fads2*, *Elovl2*, among others).

Fragmentation by tandem mass spectrometry (MS/MS) revealed that the polyunsaturated lipid species that were increased tended to contain a 20:4-fatty acyl chain. Since the rate-limiting step for in vivo generation of 20:4 is the desaturation of 18:2 by FADS2 enzyme, we determined the “Fads2 index”, as a proxy for FADS2 activity. For that, we calculated the ratio of {PC(36:4)[16:0/20:4] + PC(38:4)[18:0/20:4]} to {PC(34:2)[16:0/18:2] + PC(36:2)[18:0/18:2]} and found this to be significantly increased in the *Rnf43/Znrf3*^*del*^ mice, compared to their respective controls. Furthermore, there was a significant positive correlation between the calculated PC Fads2 index and *Fads2* gene expression from the transcriptomic data (Fig. 3e).

Next, we used mass spectrometry imaging (MSI) to visualise potential changes in lipid zonation^31^. As shown previously, we found that PC(32:0) was co-localised with the portal vein, while PC(36:4) and PC(32:1) closely recapitulated perivenous (zone3) and periportal (zone1) regions, respectively (Supplementary Fig. 4a). Interestingly, we found increased intensity as well as expansion in the spatial area covered by PC(36:4), relative to PC(32:1) and PC(34:2), in the *Rnf43/Znrf3*^*del*^ compared to WT, highlighting the expansion of the pericentral region (Fig. 3f). Finally, IF analysis of LPCAT2 (an enzyme that incorporates 20:4 into PC), revealed that the intensity and spatial area of LPCAT2 were increased in *Rnf43/Znrf3*^*del*^ (Supplementary Fig. 4b).

Taken together, the lipidomic and lipid imaging analyses point towards a clear alteration in the liver lipid composition and lipid distribution across the tissue, with a significant increase in the abundance of unsaturated lipids, likely caused by upregulated lipid desaturase enzymes.

To investigate whether part of the lipid alterations observed was due to a cell-autonomous effect of *Rnf43/Znrf3* in hepatocyte lipid metabolism, we studied the impact of deleting both genes in hepatic cells ex vivo. For that, we took advantage of liver organoid cultures that enable the expansion of mutant hepatocytes derived from mouse embryonic^32^ and adult^33^ tissue in the absence of stromal niche. Hence, we isolated *Rnf43/Znrf3*^*flox*^ hepatocytes or hepatoblasts and then deleted *Rnf43/Znrf3* to generate *Rnf43/Znrf3*^*del*^ mutant cultures and corresponding parental (non-deleted) controls (Fig. 3g and methods) and analysed their lipid content by staining for neutral lipids. In both models, we observed a significant increase in the number and size of the lipid droplets in the *Rnf43/Znrf3* mutant cultures, which was completely reverted upon treatment with WNT inhibitors (Fig. 3h, i and Supplementary Fig. 4c, d). Notably, treatment of parental WT (*Rnf43/Znrf3*^*flox*^) hepatocyte organoids with WNT3A ligand also resulted in a significant increase in the number and size of lipid droplets (Fig. 3j, k).

In summary, the combination of the transcriptional, lipidomics and lipid imaging analysis and organoid data reveals a role for RNF43/ZNRF3 as gatekeepers of the lipid metabolic ground-state in hepatocytes, through the combination of both cell-autonomous (direct intracellular accumulation) as well as indirect (e.g., microenvironmental/zonation changes) mechanisms, such as the expansion of the metabolic programmes of the pericentral hepatocytes.

### *Rnf43/Znrf3* mutant mice exhibit regeneration defects

**Fig. 4:**
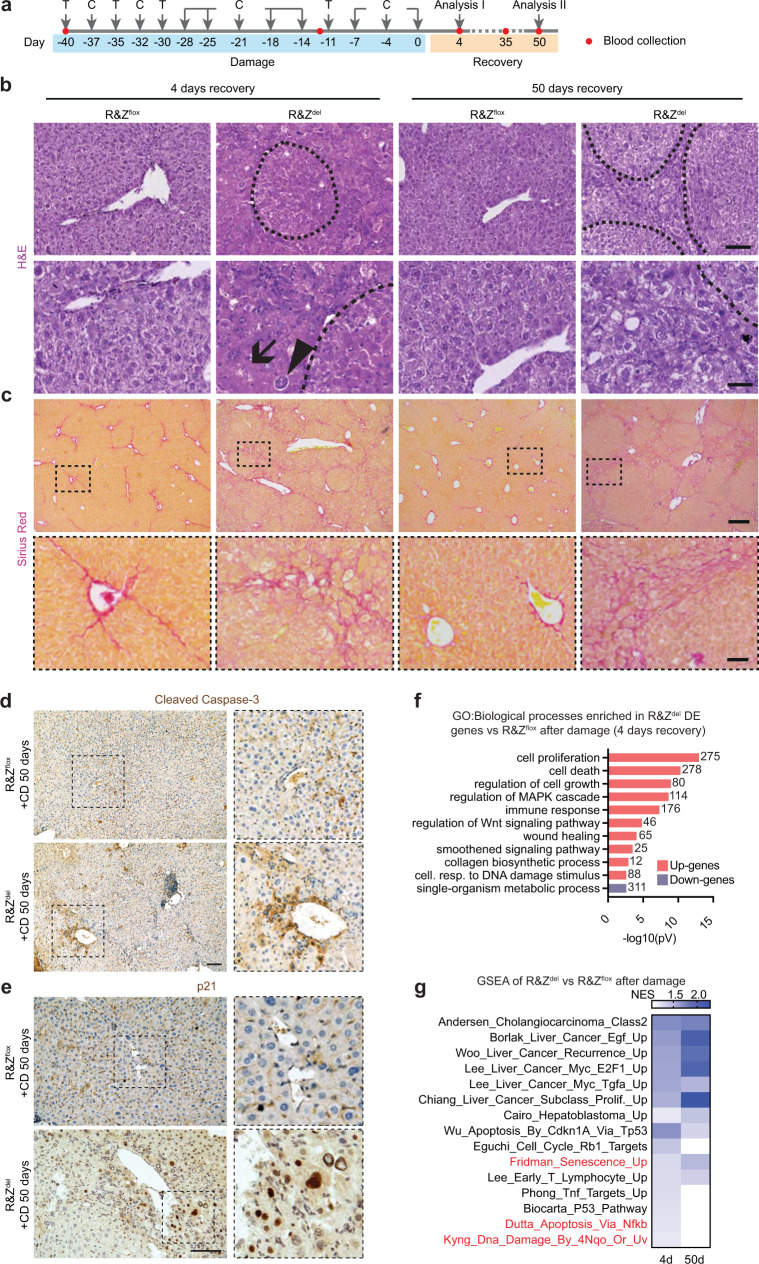
Liver-specific *Rnf43/Znrf3* deletion leads to defective tissue regeneration after chronic CCl_4_ treatment. **a**–**g** Adult *AlbCreERT2xRnf43/Znrf3*^*flox*^ mice received tamoxifen to generate *Rnf43/Znrf3* null livers. After a washout period, CCl_4_ was injected twice a week for a total of 6 weeks and mice were collected at 4, 35 and 50 days after the last injection. These time points are equivalent to 1.5, 2.5 and 3 months post recombination, and hence comparable to the 1 month and 3 months undamaged mutant mice. Tamoxifen was re-administered to prevent expansion from un-recombined hepatocytes, which ensured a >80% recombination of both alleles (See Supplementary Fig 5). **a** Experimental design. T tamoxifen, C CCl_4_. **b** Representative pictures of H&E staining of *n* = 4 (CD 4d) and *n* = 3 (CD 50d) independent replicates. Histopathological analysis revealed increased tissue damage as evidenced by the presence of giant hepatocytes (arrowhead), de-cellularised areas (arrow) and regenerative nodules (dashed line) in *Rnf43/Znrf3* mutant livers compared to littermate controls. Scale bar, 100 μm (top) and 50 μm (bottom). **c** Collagen deposition (Pico-Sirius red staining) in mutant livers after damage indicate mild fibrosis. Representative pictures of *n* = 4 (CD 4d) and *n* = 3 (CD 50d) independent biological replicates. Scale bar, 500 μm. Quantification of the fibrotic area is given in Supplementary Fig 5f. **d**, **e** Cleaved caspase-3 (**d**) and senescence marker p21 (**e**) stainings at 50 days of recovery from chronic damage. Representative images of *n* = 3 independent replicates. Scale bar, 100 μm. **f** Graph showing selected gene ontology (GO) terms significantly enriched for genes upregulated (red) and downregulated (purple) in R&Z^del^ compared to R&Z^flox^ after damage. Numbers on the bars denote the number of genes associated with each term. Full list in Supplementary Data 1_S29–S32. **g** Heatmap of the normalised enrichment score (NES) of selected GSEA data sets at 4 and 50 days of recovery. Red, gene sets of apoptosis and senescence. Full list in Supplementary Data 1_S38–S45. Source data in Source data file.

Our observations that *Rnf43*/*Znrf3* livers exhibit a significant degree of parenchymal degeneration, steatohepatitis and increased unsaturated lipids—also associated with inflammation and NASH^34^—prompted us to hypothesise that the homoeostatic turnover of the hepatic parenchyma might be impaired in *Rnf43*/*Znrf3*^*del*^ mice. Hence, we decided to investigate the role of RNF43/ZNRF3 during damage-regenerative response. For that, we challenged *Rnf43/Znrf3*^*del*^ mice with repeated doses of carbon tetrachloride (CCl_4_)^35^, in order to induce chronic liver damage (Fig. 4a and Supplementary Fig. 5a).

As a proxy for liver damage, we quantified the levels of transaminases in serum before the first dose of CCl_4_ (day −40), during damage (day −12, middle of the regimen, after 7× doses of CCl_4_) and at several time points after the last dose (day 4, 35 and 50 of recovery, which correspond to 1.4, 2.5 and 3 months post-deletion). We observed significantly higher levels of ALT, AST and ALP in our mutant mice compared to WT littermates undergoing the same damage regimen, which persisted even when control mice had fully recovered (Supplementary Fig. 5b). In addition, mutant livers were significantly bigger and presented multiple regenerative and pre-neoplastic nodules not observed in non-damage controls (Supplementary Fig. 5d, e). Histological analysis revealed hallmarks of prominent tissue damage, including decellularized regions, significant fibrosis and extensive ductular reaction (Fig. 4b, c and Supplementary Fig. 5f, g). Senescence (p21), apoptotic (cleaved caspase-3) and damage (HMGB1) markers confirmed that CCl4-treated *Rnf43/Znrf3*^*del*^ livers were extensively damaged (Fig. 4d, e and Supplementary Fig. 5h). Expectedly, we also found a significant increase in the GS staining area after 50 days of recovery, but not after 4 days, when the cells expressing this marker are highly damaged by the CCl_4_ (Supplementary Fig. 5i). These prominent tissue damage alterations (decellularization, fibrosis, cytoplasmic HMGB1 staining) were not detected in control littermates, which had mostly recovered already at day 4 after the last CCl_4_ injection (Fig. 4b–e and Supplementary Fig. 5b, d–i) neither in mutant livers not exposed to damage at the same time point (3M, Supplementary Fig. 1).

To gain further insights, we performed RNAseq analysis of the CCl_4_-damaged *Rnf43/Znrf3*^*del*^ and *Rnf43/Znrf3*^*flox*^ livers at days 4 and 50 of recovery. We found a significant number of genes DE (2989 at day 4 and 1438 at day 50) including several metalloproteases (*Mmp7, Mmp11, Mmp2, Mmp23*) and components of the caspase-mediated apoptosis signalling pathway (*Casp12*, *Pycard*) (Supplementary Data 1_S16–S19). In addition, we found an increased number of proliferative (Ki67+) cells (Supplementary Fig. 6a–b), in agreement with the reports of active β-catenin^36^ and liver-specific APC mutant mice^37^. GO term and gene set expression analysis (GSEA) confirmed that many biological processes involved in proliferation were activated at both time points, including “regulation of cell growth”, “proliferation” and “wound healing”. Interestingly, we found significant enrichment for liver cancer data sets, as well as data sets associated with tissue and DNA damage and inflammation (Fig. 4f, g and Supplementary Data 1_S38–41).

Altogether, the serum, histological and transcriptional findings indicated that at the molecular and histological level, *Rnf43/Znrf3*^*del*^ livers were still undergoing a damage-regenerative response at day 50 of recovery, when the WT tissue had fully regenerated. Notably, a single acute dose of CCl_4_ resulted in similar levels of damage in both genotypes (Supplementary Fig. 5c), suggesting that at baseline both genotypes have a similar response to damage.

### *Rnf43/Znrf3* mutants present defective proliferation/differentiation states

**Fig. 5:**
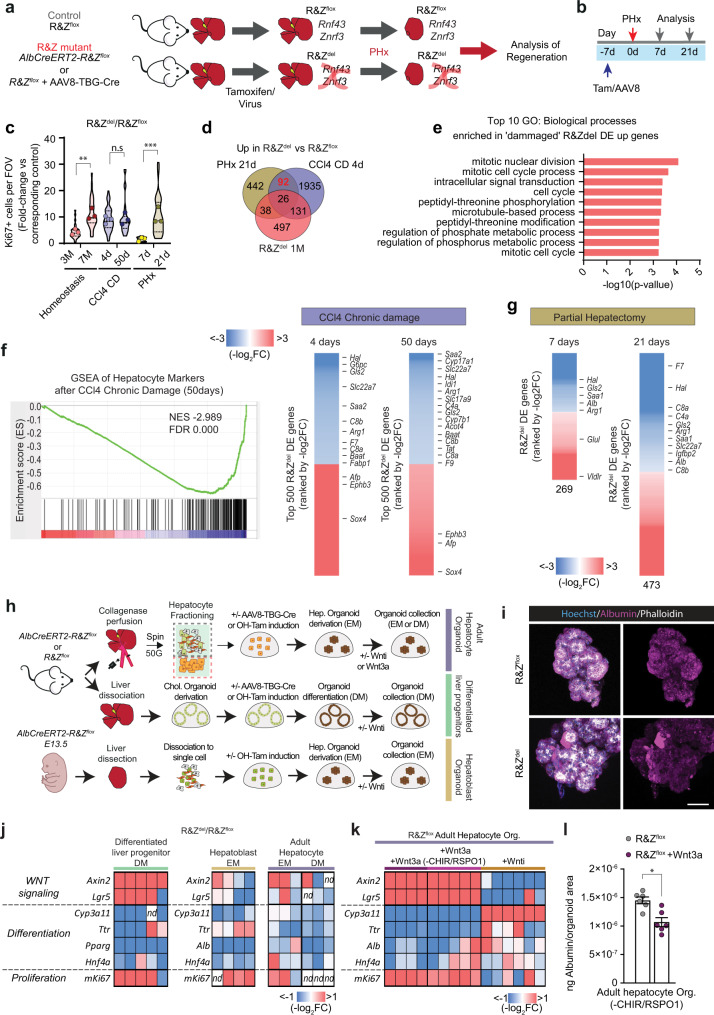
*Rnf43/Znrf3* loss impacts the regenerative capacity of hepatocytes by altering their proliferation/differentiation states. **a**–**g** Adult R&Z^flox^ mice were treated with tamoxifen or AAV8-TGB-Cre to generate R&Z^del^. Controls were treated with vehicle or AAV8-null virus. Induced mice underwent partial hepatectomy (PHx) and samples were collected at the indicated time points. **a**, **b** Experimental design (**a**) and timeline (**b**). **c** Fold change of the number of proliferating (Ki67+) cells in R&Z^del^ livers with respect to corresponding controls in homoeostasis (3 or 7 months of deletion), after CCl_4_ (4 or 50 days recovery) or PHx (7 or 21 days recovery) damage. Violin plot showing median, IQR and full distribution of a minimum of 10 FOV per mouse (*n* = 3 or *n* = 4, CD 4d). Two-way ANOVA with Sidak multiple comparisons. Homeostasis, **p = 0.0023; PHx, ***p = 0.0003. **d**, **e** Common signature of 92 genes upregulated in the mutant upon PHx and CCl_4_ chronic damage. **d** Venn diagram. **e** Top 10 GO biological processes. Details in Supplementary Data 5. **f**, **g** Hepatocyte markers are significantly downregulated in the R&Z^del^ vs WT upon CCl_4_ (**f**) and PHx (**g**) damage. **f** Left, GSEA analysis between hepatocyte signature and CCl_4_-damaged. Details in Supplementary Data 1_S37. Right, DE genes in R&Z^del^ vs R&Z^flox^ after chronic CCl_4_ treatment. Heatmaps present the Top500 (FDR < 10%) ranked by Log2FC and averaged between all mice. **g** Heatmaps of DE genes upon PHx ranked by Log2FC and *p* value <0.01 and averaged between all mice (Likelihood ratio test and Benjamini–Hochberg correction). Number, the number of genes. **h**–**l** Wild-type and R&Z^del^ adult liver ductal, hepatocyte or hepatoblasts organoids were generated as detailed in “Methods” and grown in expansion media (EM, devoid of WNT3a) or differentiation media (DM, devoid of WNT3a and RSPO1) or treated with Wnt inhibitors (Wnti) or WNT3a media before collection. Note that, for WNT3a, two conditions were used: WNT3a in expansion medium (Wnt3a) and Wnt3a in the absence of CHIR 99021 and RSPO (Wnt3a −CHIR/RSPO). **h** Experimental design. **i** Representative pictures of hepatoblast organoids stained for Albumin (magenta). **j**, **k** qPCR expression analysis on the indicated genes in R&Z^del^ vs R&Z^flox^ in differentiated cholangiocyte (left) or hepatoblast (middle) or adult hepatocyte (right) organoids grown in the indicated media (**j**) or R&Z^flox^ adult hepatocyte organoids treated or not with WNT3A or Wnti. Heatmap represents the fold change values of R&Z^del^ vs R&Z^flox^ (**j**) or R&Z^flox^ treated vs untreated (**k**). Each column is a biological replicate. nd not detected. **l** Albumin secretion in R&Z^flox^ hepatocyte organoids in WNT3A in the absence of CHIR and RSPO. Data represents mean + /− SEM from *n* = 2 replicates from *n* = 3 independent experiments. Paired two-tail *t* test was used, *p = 0.0135. Source data in Source data file.

We next studied the mechanism for defective regeneration. We investigated whether loss of *Rnf43/Znrf3*, and the subsequent WNT activation, directly impact the proliferation/differentiation state of hepatocytes or whether the impaired regeneration observed was a consequence of the damage accumulated in mutant mice with an already expanded zonation, or both. For that, we analysed the regenerative response of *Rnf43/Znrf3*^*del*^ livers upon partial hepatectomy (PHx), a model that is not zonally biased (Fig. 5a, b). Hepatectomized mutant livers presented increased numbers of Ki67+ cells at day 21 after recovery when all control livers had returned to baseline (Supplementary Fig. 6c, d). Interestingly, the fold change (FC) of the number of proliferative cells in *Rnf43/Znrf3*^*del*^ livers increased to the same levels of the mutant mice recovered from chronic damage after 50 days (Fig. 5c), supporting a scenario whereby the absence of *Rnf43* and *Znrf3* “locks” hepatocytes in a proliferative state.

RNAseq analysis on *Rnf43/Znrf3*^*flox*^ and *Rnf43/Znrf3*^*del*^ livers at 7 and 21 days after PHx revealed that many of the genes altered by the damage corresponded to molecular signatures of cell proliferation as well as liver cancer (Supplementary Fig. 6e, f), with canonical WNT signalling pathway as the top upstream regulator (Supplementary Fig. 6g). When integrating the data from both damage models (PHx and CCl4-CD) we identified 92 unique genes that were commonly upregulated only during recovery (day 21 after PHx and CD after 4 days), whose GO terms corresponded to cell cycle, proliferation and cell division, confirming that the absence of *Rnf43*/*Znrf3* results in a hyper-proliferative state in hepatocytes, regardless of the damage type (Fig. 5d, e and Supplementary Data 5).

Taken together the data suggested a model whereby *Rnf43/Znrf3* mutant hepatocytes once they activate a proliferative state cannot exit from it to complete differentiation and maturation, which, consequently, impairs the tissue to terminate the regenerative process. To investigate this hypothesis, we performed GSEA between CCl_4_-damaged *Rnf43/Znrf3*^*del*^ livers and a signature of mature hepatocytes^38,39^ (Supplementary Data 1_S37). We observed a significant negative enrichment against hepatocyte signatures, indicating that the hepatocyte cell fate was downregulated (Fig. 5f and Supplementary Fig. 6h). Detailed inspection of the significantly DE genes revealed that several well-known hepatocyte markers were significantly downregulated in CCl_4_-treated mutants compared to controls, including genes involved in the complement complex (*C8a, C8b)*, coagulation factors (*F7 and F9*) and basic hepatocyte functions (*Baat*, *Tat*, *Arg1*, *Alb*, *Hal*), among others. Notably, similar results were obtained when analysing the livers of mutant mice at 7 days and 21 days after PHx (Fig. 5g), a model of compensatory hyperplasia not associated with massive necrosis but to the activation of hepatocyte proliferation^40^. Both results combined indicate that it is the imbalance between proliferation/differentiation in *Rnf43/Znrf3* mutant hepatocytes that explains the defective termination of the regenerative programme and persistence of proliferative hepatocytes, far beyond the end of regeneration phase is terminated in WT cells.

To determine whether the differentiation capacity of the *Rnf43/Znrf3*^*del*^ hepatocytes was reduced due to a cell-autonomous effect, we studied the impact of deleting *Rnf43/Znrf3* in hepatocyte cells in vitro, in organoid cultures. For that, we deleted both genes in organoids derived from either adult hepatocytes^33^ or our organoid culture system whereby progenitors, either hepatoblasts^32^ or adult liver ductal cells differentiate into hepatocyte-like cells^41^ (Fig. 5h–l and Supplementary Fig. 6i, j). In agreement with the mouse data, *Rnf43/Znrf3*^*del*^ organoids exhibited increased proliferation and reduced differentiation capacity than control WT organoids, as assessed by quantitative polymerase chain reaction (qPCR) analysis of common hepatocyte markers such as *Ttr*, *Cyp3a11*, *Pparg*, *Alb* and *Hnf4a* and WNT target genes such as *Lgr5* and *Axin2,* or the proliferation marker *mKi67* (Fig. 5j). To investigate the direct role of WNT hyperactivation in the differentiation defect, we treated *Wild-Type* organoids with either Wnt-inhibitors (IWP2^42^ and iCRT3^43^) or WNT3A ligand, to completely inactivate or mimic the hyperactivation of the pathway. The blockade of WNT signal resulted in an increased differentiation ability of the adult hepatocytes. Conversely, supplementing WNT3A in the media promoted an undifferentiated and highly proliferative state, mimicking the *Rnf43/Znrf3*^*del*^ phenotype (Fig. 5k, l). Similarly, Wnt inhibition of mutant organoids also partially rescued the differentiation defect (Supplementary Fig. 6j). As expected, Wnt-inhibition treatment abolished the expression of the Wnt targets *Lgr5* and *Axin2*, while Wnt3a upregulated them (Fig. 5k).

Collectively, these results imply that loss of RNF43/ZNRF3 activates the proliferative programme of hepatocytes while at the same time impairs their ability to terminate differentiation, which combined with the extensive tissue degeneration and changes in the cellular microenvironment, could lead to eventual liver fibrosis and the degenerative tissue phenotype observed upon chronic injury.

### RNF43/ZNRF3 deletion predisposes to liver cancer

**Fig. 6:**
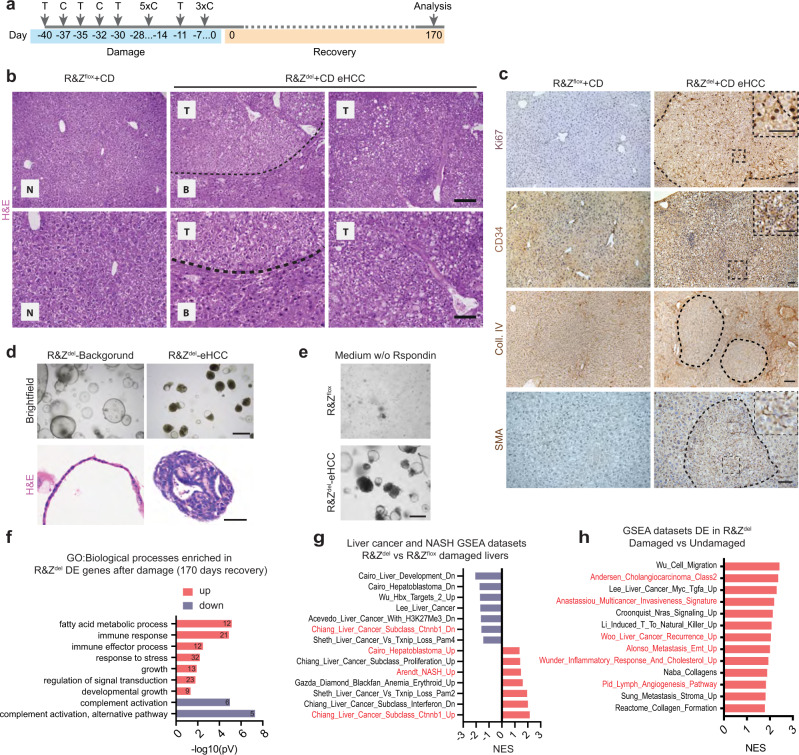
Liver-specific *Rnf43* and *Znrf3* (R&Z) deletion leads to early hepatocellular carcinoma after chronic damage. **a** Timeline of the experiment. T tamoxifen, C CCl_4_. **b**, **c** Histopathological analysis revealed the presence of early hepatocellular carcinomas. Dashed black lines mark eHCC border. **b** Representative pictures of six different biological replicates of H&E stainings of tumours and background tissues. N normal tissue, B background tissue, T tumoural tissue. Scale bar, 200 μm (top panel); 100 μm (bottom panel). **c** Tumours were characterised by increased proliferation (Ki67), vascularisation (CD34), disruption of tissue architecture (Collagen IV, Coll.IV) and stromal invasion (SMA). Representative images of at least four different samples analysed are shown. Scale bars, 100 μm. Dashed black lines mark eHCC border. **d** Brightfield and H&E staining representative images of tumouroids isolated from an eHCC lesion. Isolation of tumouroids was repeated three times. Scale bar, 500 μm (brightfield) and 50 μm (H&E). **e** Brightfield pictures of organoids cultured without R-spondin in two independent experiments. Scale bar, 200 μm. **f** Graph showing selected gene ontology (GO) terms significantly enriched for genes upregulated (red) and downregulated (purple) in R&Z^del^ compared to R&Z^flox^. The numbers denote the number of genes associated with each term. The full list of significant terms can be found in Supplementary Data 1_S33–S34. **g**, **h** Graphs show normal enrichment score (NES) for selected GSEA data sets significantly enriched (FDR < 25%; *p* < 0.05). **g** GSEA of the DE genes in R&Z^del^-damaged vs WT-damaged (R&Z^flox^) at 170 days recovery. The full list can be found in Supplementary Data 1_S42–S43. **h** GSEA of the DE genes in R&Z^del^-damaged livers at 170 days of recovery vs R&Z^del^ undamaged livers at the same time point post deletion (7M). The full list can be found in Supplementary Data 1_S46–S47. Source data are provided as a Source data file.

To assess whether the steatohepatitis/lipotoxicity and malfunctioning regenerative capacity of *Rnf43/Znrf3*^*del*^ mice would progress into a malignant HCC we subjected the mice to chronic injury and collected the livers ~5 months later (170 days after the last dose of CCl_4_, 7 months after deletion) (Fig. 6a). We noted the appearance of tumoral nodules of different sizes already at the macroscopic examination of the tissue (Supplementary Fig. 7a). Histopathological analysis confirmed that these were HCC (HCC) or HCC with ductal features (CHC, mixed subtype), with a combined penetrance of 89% (8 out of 9 mice) (Fig. 6b and Supplementary Fig. 7b–d). HCC lesions were characterised by small cells with high nuclei/cytoplasmic ratio, a compact pattern without the trabecular formation and 2-3 cell thick plates, fat accumulation, a significant number of proliferative (Ki67^+^) cells, neo-vascularisation and disruption of tissue histoarchitecture as evidenced by CD34 staining, Collagen IV deposition and stroma invasion (SMA+ cells). Additionally, the tissue presented increased fibrosis, proliferation and ductular reaction as assessed by Oil red-O, Sirius red, Ki67 and PCK staining, respectively (Fig. 6b, c and Supplementary Fig. 7c–h). The mixed HCC (CHC) lesions in addition presented glandular structures formed by PCK^+^ ductal cells (Supplementary Fig. 7d). None of these malignant features was observed in any of the damaged-WT controls (Fig. 6b, c and Supplementary Fig. 7a–h) while, as mentioned, only a little proportion of undamaged mutant mice (10%, 2 out of 20) presented adenomas at this time point post-deletion (Supplementary Fig. 2b).

To better characterise the HCC lesions, we microdissected the tumour and either processed them for molecular analysis or cultured them in our previously described human primary liver cancer organoid method^44^. Molecular analysis indicated that the HCC tumours were indeed devoid of *Rnf43/Znfr3* and expressed high levels of *Axin2*, confirming the *Rnf43/Znrf3*^*del*^ origin of the tumour lesion (Supplementary Fig. 7i). Interestingly, HCC lesions but not the background liver expanded as tumour organoids in vitro by forming compact structures composed of tumoural cells that filled in the lumen of the organoid and expressed high levels of WNT target genes (*Axin2* and *Sp5*) (Fig. 6d and Supplementary Fig. 7j). Of note, *Rnf43/Znrf3*^*del*^ tumour organoids, but not WT controls, grew long-term (up to passage 10) in the absence of RSPO1, confirming their origin from *Rnf43/Znrf3*^*del*^ cells (Fig. 6e).

RNAseq analysis identified 214 DE genes between mutants and WT littermates subjected to the same regimen and recovery time. GO and GSEA analysis revealed enrichment on fatty acid metabolism, and several cancer signatures, including tumours with β-catenin activation^45^, hepatoblastoma^46^, cancer patients with NASH^47^ as well as advanced cancer and metastatic cancer data sets, and inflammation and cholesterol uptake data sets (Fig. 6f–h and Supplementary Data 1_S18). Similar enrichment was also detected at an earlier time point (at 50 days recovery) (Supplementary Fig. 7l).

Altogether, this data indicated that RNF43/ZNRF3 loss predisposes to cancer due to altered lipid metabolism and defective tissue regeneration (hepatocyte maturation) upon cumulative rounds of tissue damage.

### *RNF43*/*ZNRF3* patients present altered lipid metabolism and poorer prognosis

**Fig. 7:**
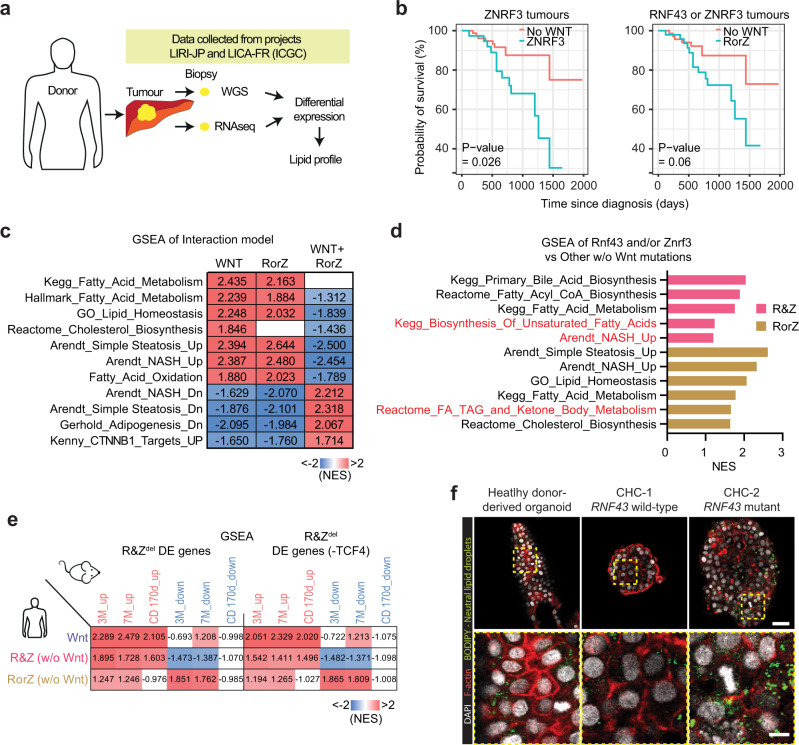
Human HCC patients mutated in RNF43 and/or ZNRF3 present lipid metabolic alterations and poor prognosis. **a**–**e** Clinical data, whole genomic sequencing and RNAseq data were downloaded from ICGC database and used to determine the prognosis and expression pattern of human tumours mutated in *RNF43* and/or *ZNRF3* or other WNT pathway components (*APC*, *AXIN1* or *CTNNB1*). Full details in “Methods”, Supplementary Fig. 8 and Supplementary Data 6. **a** Experimental design. **b** Survival analysis after Cox proportional hazards model found a significant interaction with gender, tumour stage and WNT mutation status. All covariates passed the proportional hazards test except WNT mutation. The graph shows the survival analysis of HCC patients with ZNRF3 (ZNRF3, left) or RNF43 or ZNRF3 (RorZ, right) mutations compared to patients with no-WNT mutations (No-WNT). *p* Values obtained by Wald statistic, CI 95%. **c**, **d** Differential gene expression for patients with RorZ and R&Z mutations compared to non-WNT mutants (other w/o Wnt mutation). **c** A multiple regression model indicated an interaction between WNT mutation status and the signatures of *RNF43* or *ZNRF3* patients. Note that the presence of WNT mutation (WNT + RorZ) negatively impacts the expression of RorZ patients, with gene sets positively enriched in RorZ mutants becoming negatively enriched. **d** Significantly enriched (*p* value <0.05, two-sided permutation test) gene sets in R&Z and RorZ-mutated patients compared to patients with no-WNT mutations. **e** Comparison between the mouse differentially expressed genes including or excluding TCF4 targets and the signatures of patients with mutations in R&Z, RorZ and WNT (*APC*, *CTNNB1* or *AXIN1*). The table represents the GSEA (NES) of the DE genes in R&Z^del^ (3 and 7 months homeostasis or 170 days after damage, including or excluding TCF4 targets) against WNT, R&Z or RorZ human liver tumour signatures. NES scores are presented as a heatmap where red is positively enriched and blue is negatively enriched. Note the significant correlation. **f** Human liver organoids from healthy donors or patients with liver cancer (mixed subtype, CHC and mutations or not in RNF43) were grown and stained for neutral lipids (Bodipy, Green). Representative images from *n* = 2 independent experiments. Red, F-actin staining. White, Dapi. Scale bar, 60 μm (top panel); 15 μm (bottom panel).

To determine the relevance of our findings in human liver cancer, we next assessed the effect of *RNF43/ZNRF3* mutations in human primary liver tumours taking advantage that the projects LIRI-JP and LICA_FR from the ICGC collection^48,49^, contained genomic and expression data of the same tumours (Fig. 7a). Within the somatic variant mutations that passed our filtering criteria, we found mutations in both coding as well as non-coding regions (mainly intron variants, see methods and Supplementary Data 6_S1). We used these patients to study the prognostic value of the two genes, for those samples where survival and clinical information was available (*n*  = 257 donors from LIRI-JP data set, “Methods”). Using multivariate survival analysis accounting for WNT mutation, *RNF43 or ZNRF3* mutation, double *RNF43 /ZNRF3* mutation, donor sex and clinical tumour stage, we observed a significant difference in the effect of *RNF43* or *ZNRF3* mutation on survival depending on whether or not a mutation in WNT was also present (interaction term *p* value 0.049, Supplementary Data 6_S13). Hence, we next considered the effect of *RNF43/ZNRF3* conditional on WNT mutation status. We separated the groups by those containing WNT mutations and those not (*-CTNNB1*, *APC*, *AXIN1* mutations, see “Methods”). Interestingly, for those without WNT mutations, we observed that patients bearing ZNRF3 tumours had a significantly poorer prognosis than patients with WT tumours (*p* = 0.026). This result was independent of clinical features, sex and tumour stage. A similar trend was observed when evaluating patients harbouring either *ZNRF3* or *RNF43* mutations, although this was not statistically significant (*p* = 0.06) (Fig. 7b and Supplementary Data 6_S14–S15). Conversely, neither *ZNRF3* alone nor *ZNRF3* or *RNF43* showed altered prognosis when patients had WNT mutations (Supplementary Fig. 8b). For single *RNF43* or double mutations, the number of patients was too low to provide meaningful conclusions. The effect of WNT mutation across all patients was not significant (*p* value 0.32) consistent with what had been previously reported^21^ (Supplementary Data 6_S13). Of note, we did not find reported liver cancer driver genes shared between all *RNF43/ZNRF3* patients (Supplementary Data 6_S12).

We next performed differential expression analysis of the livers of patients harbouring WNT mutations (WNT), *RNF43* and *ZNRF3* (R&Z) and *RNF43* or *ZNRF3* (RorZ*)* compared to liver cancer patients with WT alleles for these. Among the DE genes, we found lipid metabolic genes, including *LPCAT2* and *APOA4* (Supplementary Data 6_S16–S18). GSEA analysis of the DE genes for the *RNF43* and/or *ZNRF3* groups revealed enrichment in lipid metabolism. In addition, the group containing only *RNF43* or *ZNRF3* mutations was also enriched on gene sets of “NASH”, “simple steatosis”, “Fatty acid metabolism”, “cholesterol biosynthesis” and “adipogenesis” (Supplementary Fig. 8c and Supplementary Data 6_S25–S30). Interestingly, we noted that the group of patients bearing WNT mutations, but not mutations in *RNF43* or *ZNRF3*, also had a significant enrichment in some of these lipid metabolic pathways (Supplementary Fig. 8c, WNT). Hence, we investigated the potential interaction between WNT mutations and *RNF43* or ZNRF3 using a multiple regression model. The results indicated that there is an impact of WNT mutations in the expression pattern of *RNF43* or ZNRF3 (Fig. 7c, compare RorZ vs RorZ+Wnt, Supplementary Data 6_S21–S23), similar to our observation with the survival analysis. Hence, to gain an understanding of the impact of *RNF43*/*ZNRF3* in lipid metabolism in human patients without the cofounding factor of other WNT mutations, we re-analysed the differential expression of *RNF43* and/or *ZNRF3* against WT patients not having mutations in WNT pathway components (*CTNNB1*, *APC*, *AXIN1*). Among the upregulated genes, we found genes involved in fatty acid synthesis (*FASN*), synthesis of glycerophospholipids (*LPCAT3*) and phospholipids (*PLA2G2A* and *PLA2G2D*) (Supplementary Data 6_S19–S20). Additionally, three extra gene sets including the “unsaturated fatty acids”, “Acyl-CoA biosynthesis” and “NASH” were now also significantly enriched in the patients harbouring mutations in both genes (*RNF43* and *ZNRF3* group) (Fig. 7d). Notably, those DE genes were enriched in TCF4 targets, with the patients bearing double mutations exhibiting a stronger enrichment compared to the patients bearing single mutations, when compared to non-WNT mutant patients (Supplementary Fig. 8d). These results were in agreement with our mouse transcriptomics (Figs. 2 and 6g and Supplementary Fig. 3a) and lipidomics data (Fig. 3). Further, we found a strong significant enrichment between the human signatures and the DE genes in our mutant mice (Fig. 7e), when considering both canonical WNT signature (TCF4 targets) as well as TCF4-independent signatures, suggesting that WNT and additional, non-WNT mechanisms may contribute to the mutant phenotype, both in human and mouse.

To investigate whether lipid accumulation was also cell-autonomous in cancer patients, we analysed the lipid droplet content in human liver cancer tumour organoids mutated in these genes. In our collection, only one line (CHC2 line)^44^ harboured a homozygous (deleterious) *RNF43* mutation (Leu418Met). We found that RNF43 mutant organoids accumulated intracellular lipid droplets, while organoids derived from liver cancer patients without mutations in any of the two genes (CHC-1) or donor-derived organoids (derived from non-cancer tissue) presented fewer intracellular lipid droplets (Fig. 7f). These results suggested that, at least in part, lipid droplet accumulation is cell-autonomous in human patients.

In summary, our results indicated that human liver cancer patients bearing *ZNRF3* and/or *RNF43* mutations present altered liver lipid metabolism, in agreement with our findings in the *Rnf43/Znrf3* mutant mouse model, and identified *ZNRF3* and/or RNF43 as potential prognostic biomarkers for liver cancer.

## Discussion

Cancer genomic studies have identified *RNF43* and *ZNRF3* as WNT pathway genes mutated in liver cancer^21,22,50^. However, their implication in liver regeneration and or liver disease remained unexplored. Here we find that loss of *RNF43/ZNRF3* predisposes to liver cancer by controlling liver lipid metabolic ground-state and the balance between proliferation/differentiation in hepatocytes. Hence, both homologues behave as landscaper genes, which contribute to neoplastic transformation by altering the tissue environment^51^. *Rnf43/Znrf3* mutant mice presented steatohepatitis and aberrant liver lipid composition, with an increase in unsaturated lipid species, in the absence of dietary fatty acid supplementation. Concomitantly, mutant livers exhibited increased proliferation and impaired regeneration capacity, which following chronic injury, progressed to liver cancer, both HCC and mixed eHCC/CC.

Our findings might seem at odds with reported results whereby WNT3A injection prevents lipid accumulation in mice with active non-canonical WNT through an LRP6 receptor mutation (LRP6^R611C^)^52,53^ and with the phenotype of the core canonical WNT component APC in the liver, which results in HCC without the need of any additional hit or tissue damage^37^. However, recent reports have demonstrated a link between WNT signal strength and pathway outcome, suggesting that the strength of activation of the pathway as well as the nuclear beta-catenin fold change, rather than absolute levels, are critical for target gene activation^54^, stem cell behaviour or malignant transformation^55–57^. Our studies where WT organoids are supplemented with WNT3A ligand (Figs. 3j, k and 5k, l) suggest that the lipid droplet accumulation and defective regeneration of the mutant mice is the result of increased sensitivity to WNT activation, which in vivo could be mediated by an increased sensitisation of mutant hepatocytes to WNT ligands secreted by the cellular microenvironment, as suggested by Sun et al.^24^. However, our organoid studies in mutant organoids (Fig. 5j) and mutant cancer organoids (Fig. 6e) show that mutated cells proliferate in the absence of WNT (EM) and WNT and RSPO ligands (DM condition and cancer organoids). This observation prompts us to an additional --yet complementary-- interpretation; in vitro, RNF43/ZNRF3 mutant cells exhibit an intrinsic, cell-autonomous activation of their proliferative state and a reduced differentiation capacity (Figs. 3h, i and 5i, j). These results combined with our observations that the 7 months mutant livers present signs of tissue degeneration (necro and apoptosis) suggest a scenario whereby the cooperation between different mechanisms, increased susceptibility to WNT ligand from the cell microenvironment, intrinsic changes to the differentiation and metabolic states and different susceptibility to rounds of damage, explains the lipid metabolic alterations and the defective hepatocyte differentiation and regeneration, which would eventually lead to increased susceptibility to liver cancer.

The significant resemblance between the mouse and human expression signatures, combined with the altered lipid metabolism and potential as prognostic biomarkers, suggests that our mouse findings are relevant to explain the pathogenesis of the disease in ZNRF3/RNF43 mutant patients. Hepatic long-chain fatty acid composition has been linked to inflammation and NASH development^34,58^. Both mice and patients present increased signatures of unsaturated fatty acid biosynthesis and NASH. This is in contrast to Sun et al.^24^, who observe increased fatty acid signatures, adipogenesis and cholesterol but not steatohepatitis in the mutant mice. The reason for this difference is unknown but is likely due to differences in genetic backgrounds or time points of analysis (in Sun et al., mice are analysed at the tumour stage, while our mice are analysed earlier and prior to tumour formation). Interestingly, both genes need to be depleted in mice to account for the development of liver cancer; however, in humans, a single *ZNRF3* mutation was a significant prognostic biomarker. A similar difference also occurs in colon cancer. The cause for this is unknown. However, it is interesting to note that Sun et al., found increased hepatocyte proliferation upon single deletions of ZNRF3 or RNF43^24^, supporting the conclusion that also single mutants could play a role in liver cancer.

Several outstanding questions arise from our investigations. First, we found a clear interaction between mutations in WNT (*APC*, *AXIN*, *CTNNB1*) and *RNF43/ZNRF3* mutations, both in prognosis and gene expression, supporting the notion that WNT and ZNRF3/RNF43 mutations might be acting through slightly different mechanisms. Both types of patients exhibited clear canonical WNT activation and lipid metabolic alterations; however, only *RNF43/ZNRF3* with no other WNT mutations (*APC/AXIN/CTNNB1*) present poorer survival, suggesting that additional non-WNT mechanisms might contribute to tumorigenesis in RNF43/ZNRF3 patients. In that regard, the transcriptomic analysis excluding TCF4 targets indicates that both, mouse and patients present non-TCF4 (non-canonical WNT) signatures that contribute to tumorigenesis. Along these lines, a recent report demonstrates a non-WNT role for RNF43 in DNA-damage response in gastric cancer cells^59^. Second, one intriguing observation from the human studies was that many of the somatic variant mutations for *ZNRF3*, contrary to *RNF43*, were in non-coding regions. Variants in introns can affect splice sites and also cause loss of regulatory repressor elements, as described for BRCA2 in Fanconi anaemia^60^. Third, we found no significant differences in the expression of any of both genes in mutant patients (Supplementary Data 6_S44), suggesting that, in humans, the mechanism is not related to transcriptional changes but potentially changes in protein function. In that regard, at least for RNF43, point mutations can result in dominant-negative forms^61–63^. Hence, our results call for future studies to assess the effect of individual *RNF43/ZNRF3* mutations in human liver tumorigenesis.

In summary, our studies provide a framework to start to understand the role of the tumour suppressors *RNF43/ZNRF3* in liver cancer. We find that the combination of mechanisms including (1) intrinsic predisposition to intracellular fat accumulation, (2) a disrupted liver lipid composition and zonation, (3) an increased hepatocyte proliferation and reduced differentiation capacity with subsequent inability to terminate regeneration and (4) an increased sensitivity to WNT ligand, they all coalesce in the perfect mix to promote malignancy. More studies are needed to characterise the role of RNF43/ZNRF3 in NAFLD development and cancer progression. With the alarming increase in the consumption of fat and sugar worldwide, recognising those individuals with mutations in these E3 ligases or any other canonical WNT signalling component might facilitate the discovery of populations at risk of developing liver steatosis, NASH, cirrhosis or cancer, and facilitate their therapeutic intervention and management of the disease.

## Methods

### Animals

#### Husbandry and gene recombination

All mouse experiments were conducted in accordance with procedures approved by the UK Home Office relating to the use of animals in research (Animals Act 1986). All mice were maintained under standard dark/light cycle, temperature and humidity conditions and both male and female adult (8-16 weeks old) mice were used in this study. Conditional knockout (KO) mice for *Rnf43* and *Znrf3* (*Rnf43/Znrf3*^*flox*^) were previously generated and described in Koo et al.^18^. Briefly, exons encoding for the ring domain of *RNF43* and *ZNRF3* were flanked by LoxP sites to drive excision in presence of a CRE protein. *Rnf43/Znrf3*^*flox*^ mice were also crossed with either Alb1-CreERT2^25^ or Sox9-CreERT2 (Jackson Laboratory JAX—http://jaxmice.jax.org/) to generate Alb1-CreERT2-R&Z^flox^ (used for the in vivo studies) and Sox9-CreERT2-R&Z^flox^ (used to generate R&Z^del^ organoids from liver progenitors). The Cre enzyme was induced by intraperitoneal (IP) injection of 200 mg/kg tamoxifen (Sigma-Aldrich) dissolved in sunflower oil. Sunflower oil only was injected to R&Z^flox^ mice, as littermate controls. Alternatively, deletion was performed after tail vein injections of 2.5 × 10^11^ vg/ml of AAV.TBG.PI.Cre.rBG. Control mice received the same dose of pAAV.TBG.PI.Null.bGH. AAV8 viruses were diluted in sterile phosphate-buffered saline (PBS) to a final volume of 100 μl. AAV.TBG.PI.Cre.rBG and pAAV.TBG.PI.Null.bGH were a gift from James M. Wilson (Addgene plasmid # 107787; http://n2t.net/addgene:107787; RRID:Addgene_107787 and Addgene plasmid # 105536; http://n2t.net/addgene:105536; RRID:Addgene_105536).

#### Liver damage models

For the chronic liver damage model, mice received IP injections of CCl_4_ (Sigma-Aldrich) at 0.5 μl/g (diluted in corn oil) multiple times according to the protocol (Figs. 4a and 6a). For the acute liver damage model, mice received a single IP injection of a lethal CCl_4_ (Sigma-Aldrich) dose diluted in corn oil at 2 μl/g (diluted in corn oil). In both cases, unrecombined, control littermates, received corn oil vehicle only.

Partial hepatectomy was performed as described in ref. ^64^ and mice were allowed to recover for 7 or 21 days before liver collection. In all cases, mice were sacrificed by either cervical dislocation or carbon dioxide overdose. Blood was collected by cardiac puncture or tail vein prick and samples were submitted for the analysis of liver transaminases levels (Clinical Pathology Laboratory, The Queen’s Veterinary School Hospital, Cambridge, UK).

### Primary hepatocyte isolation, FACS sorting and analysis

For adult hepatocyte organoid cultures, mice were culled by cervical dislocation and isolation of primary hepatocytes was performed by liver perfusion as described in ref. ^65^. For hepatocyte collection and cell sorting of the different cell compartments, mice were culled by carbon dioxide intoxication and the head was removed to allow exsanguination. Livers were perfused with 10 ml of perfusion buffer (0.5 mM EGTA in PBS), then with 10 ml of perfusion buffer with bovine serum albumin (BSA; Sigma-Aldrich) and then with 10 ml of pre-warmed 0.5 mg/ml Collagenase A (Sigma-Aldrich) solution (HBSS 1×, 5 mM CaCl_2_, 20 mM HEPES). After 2 min of incubation, livers were carefully removed, place in STOP solution (HBSS 1×, 5 mM CaCl_2_, 20 mM HEPES, 0.5% BSA) and disaggregated using tweezers. The mixture was washed several times in STOP solution (50 G for 2 min) and filtered with a 100 μm strainer. The pellet containing hepatocytes was resuspended in RLT lysis buffer (Qiagen) and frozen at −80 °C for RNA extraction. The supernatant was centrifuged again at 300 × *g* for 5 min to collect non-parenchymal cells. Pellet was divided into two fractions and stained either with EpCAM-APC (eBioscience) and CD31-PE-Cy7 (AbCAM) antibodies or EpCam-APC and CD11b-PE-Cy7 (BD Bioscience) antibodies (see Supplementary Data 7). Positive cells were sorted using a Flow Sorter MoFlo (Dako Colorado, Inc.), resuspended in RLT lysis buffer and stored at −80 °C. For analysis of the immune compartment by flow cytometry, livers were isolated and digested as previously published^41^. Single cells were subsequently stained with the corresponding labelled antibodies (Supplementary Data Set 5, 30 min, ice) before analysis in a SONY SH800S cell sorter. Liver single cells were selected by forward/scatter exclusion and the different immune cell types were determined according to the expression of the described cell markers using FlowJO_V10 (Supplementary Fig. 1g).

### Histology

Tissues were dissected and then fixed overnight in 10% buffered formalin (Sigma-Aldrich). For paraffin embedding, tissues were dehydrated in increasing concentrations of ethanol (70, 95 and 100% for 2 h each) ending in xylene and then incubated overnight in paraffin (Sigma-Aldrich). The following day, tissues were embedded in paraffin blocks and subsequently sectioned at a thickness of 5 μm using a microtome. Sections were mounted onto SuperFrost Plus slides (ThermoFisher) and incubated from 3 to 12 h at 60 °C before staining. H&E (Sigma-Aldrich), Oil Red O (Sigma-Aldrich) and PicroSirius Red (AbCAM) staining were performed according to the manufacturer’s instruction (Supplementary Data 7). For immunohistochemistry, sections were deparaffinised in two changes of xylene, 5 min each and rehydrated in descending grades of ethanol (100% 2×, 95%, 70%), 3–5 min each ending in water. Antigen retrieval was performed either enzymatically or heat-mediated based on primary antibody (see Supplementary Data 7). For enzymatic retrieval, sections were incubated 10 min at 37 °C and 10 min at room temperature (RT) with TE buffer containing 0.5% Triton X-100 and 1/1000 proteinase K (800 U/ml). For heat-mediated antigen retrieval, samples were incubated in either citrate buffer (10 mM Sodium Citrate, pH 6) or Tris-EDTA buffer (10 mM Tris base, 1 mM EDTA solution, 0.05% Tween 20, pH 9.0), heated in a microwave to 95–98 °C for 20 min and then cooled at RT for 20 min. Afterwards, samples were blocked and permeabilized for 1.5 h using TBS buffer containing 2% foetal bovine serum (FBS), 1% bovine serum albumin (BSA) and 1 or 0.1% of Triton x-100 based on antibody (see Supplementary Data 7 for details). Samples were then incubated overnight with primary antibody at an antibody-dependent concentration (see Supplementary Data 7). The following day, samples were washed in TBS and endogenous peroxidases were inactivated by incubating for 15 min in methanol containing 3% of hydrogen peroxide. Poly-HRP-GAMs/Rb IgG (Immunologic) secondary antibody was incubated for 30 min (Supplementary Data 7). After washing, the signal was developed using Bright Dab substrate kit (Immunologic) using the manufacturer’s instruction. Sections were counterstained in haematoxylin for 5 min, dehydrated and mounted using xylene-based DPX mounting media (ThermoFisher). Slides were analysed using optical microscopy. Pictures were taken in a DM4 Leica microscope and images were quantified in ImageJ (Fiji) by a researcher that was blinded to the experimental cohorts. H&E samples were evaluated for NAS score as previously described^29^.

For OCT embedding, livers were previously cryopreserved in 30% sucrose overnight. OCT blocks were subsequently sectioned at 6 μm in a cryostat for immunofluorescence staining of the corresponding antibodies (see Supplementary Data 7). Briefly, sections were washed in PBS and incubated in blocking solution (2% NDS, 1% Triton x-100 and 5% DMSO in PBS) for 2 h at 4 °C before staining with the corresponding antibodies (see Supplementary Data 7) overnight at 4 °C. After 3 washes with PBS, fluorescent-labelled secondary antibodies (see Supplementary Data 7) were eventually added in blocking buffer for 2 h at RT. Nuclei were stained by Hoechst 33342 (Invitrogen) 1:1000 in PBS for 5 min before mounting in Vectashield mounting medium. Slides were analysed in an Olympus Fluoview FV1000.

### Lipidomics analysis

Lipids were extracted from liver tissue using the Folch method. Briefly, liver tissue was homogenised in chloroform:methanol (2:1, 1 ml) using TissueLyzer (Qiagen Ltd., Manchester, UK). Deionized water (400 μl) was added, and the samples were well mixed. Separation of the aqueous and organic layers was carried out following centrifugation (12,000 × *g*, 10 min). The lipid-containing organic extract was evaporated in a vacuum centrifuge, without heat, and stored at −80 °C until analysis. Details on lipidomics analysis can be found in Supplementary Methods.

### Mass spectrometric imaging

Ten-μm frozen liver sections were placed on glass microscope slides (VWR) using a cryostat. Adjacent sections were stained with H&E (Sigma Aldrich). Matrix solution (5 mg/ml) of norharmane (Sigma‐Aldrich, St. Louis, MO) was prepared in 2:1:0.8 methanol:chloroform:water (v/v) and administered to the tissue surface using a SMALDIPrep sprayer (TransMIT GmbH, Giessen, Germany). Imaging experiments were performed using an AP-SMALDI10 source (TransMIT GmbH) coupled to an Orbitrap Q-Exactive (Thermo Fisher Scientific) at 10 μm increments across the tissue, using 2D-line mode. Spectra were acquired in positive ion mode from 250 to 1000 *m*/*z* at 70,000 resolution. Data were converted to imzML format^66^ and analysed using MSiReader v1.0^67^. Lipid identity was performed by accurate mass using LIPID MAPS® structure database (<5 p.p.m.) (https://www.lipidmaps.org/data/structure/).

### RNA extraction, reverse transcription–PCR and qPCR

RNA was extracted from tissues, cells or liver progenitor organoid cultures using the RNeasy Mini RNA Extraction Kit (QIAGEN) according to the manufacturer’s protocol except in the case of hepatocyte organoids where RNA was extracted using the Arcturus PicoPure RNA Isolation Kit (Thermo Fisher Scientific). In all cases, RNA was reverse transcribed with the Moloney Murine Leukaemia Virus reverse transcriptase (M-MLVRT) (Promega) as follows: 10 min at RT, 50 min at 50 °C and 15 min at 70 °C. The resulting cDNA was amplified with the iTaq™ Universal SYBR. Green Supermix (Bio-Rad) and a desired primer pair on the CFX Connect™ Real-Time PCR Detection System (Bio-Rad) and Bio-Rad CFX Manager software. The cycling conditions used were: 95 °C for 3 min and 39 cycles of 95 °C for 10 s and 55–60 °C for 30 s (depending on the primer pair), followed by a melting curve (from 55 to 60 °C to 95 °C with an increase of 0.5 °C per cycle) to ensure amplicon specificity. Gene expression was normalised to the expression of the housekeeping gene Hprt. Primers used are listed in Supplementary Data 7.

### RNAseq and analysis

RNA libraries were prepared for sequencing using the Smart-Seq2 protocol. Details on sequencing and analysis can be found in Supplementary Methods.

#### TCF4-binding analysis

The location of TCF4 peaks was taken from a previous study^15^ and annotated using ChIPseeker (version 1.12.1)^68^ in R on the mm9 genome. Genes were filtered to include those with a peak within 5 kb upstream or downstream of their transcriptional start site. We defined a lipid metabolism set of genes by combining genes contained in the GSEA hallmark fatty acid metabolism gene set, the Reactome cholesterol biosynthesis gene set, Wang classic adipogenic targets of Pparg and the GO lipid metabolic process (including child terms) set. Random peak sets were generated by shuffling the peak locations using ChIPseeker. This was done 1000 times to generate empirical *p* values for the enrichment of lipid metabolism genes with TCF4-binding sites. Venn diagrams were generated using the web tool Venny 2.1 (http://bioinfogp.cnb.csic.es/tools/venny/).

#### RNAseq differential expression analysis

For the homoeostasis model (R&Z^del^ vs R&Z^flox^ at 1 month, 3 months and 7 months of deletion), reads were filtered to keep genes with counts per million >1 in at least 3 samples. A multiple regression model was fitted with three terms, one a factor representing the condition (WT or KO) and the time-point of the sample (1 month, 3 months or 7 months), one denoting the sex of the mouse and the third denoting the batch of the sample as the data were generated in two separate batches. Normalisation was done using calcNormFactors and variance estimators calculated using estimateDisp in edgeR. Differential expression between WT and KO mice was calculated by fitting the appropriate contrast. For example, a contrast of the difference between KO at 1M and WT at 1M. Significantly DE genes for GO and GSEA were defined as having a false discovery rate (FDR) <10% and an absolute log_2_ FC >1 (Supplementary Data 1). For TCF4 and lipid metabolic analysis differential expression of R&Z^del^ livers was defined as *p* value <0.05 and an absolute log_2_ FC >0.4 to increase the depth of the analysis (Supplementary Data 3). Empirical *p* values for enrichment of TCF4 regulated genes in those showing differential expression was similarly calculated using the random peak sets used in the TCF4 peak analysis.

For partial hepatectomy differential expression analysis, a PCA plot was drawn following rlog transform (DESeq2) for all samples. The PCA plot was coloured according to the genotype and time-point and split the data on the first principal component. For the differential expression analysis, all 24 samples from the PCA analysis were used. This comprised 8 mice that had undergone damage and 6 non-damage mice at 1 month included as additional controls. The model included a term relating to the condition (either WT or KO) and the time point (either 7 or 21 days for the damaged mice and 1M for the non-damaged). This combined condition and time point factor also accounts for the difference between damage and non-damage as all non-damage mice were at 1M. Based on the PCA results, a factor for the sex of the mouse was also included in the regression model. Ensembl IDs were mapped to HUGO gene symbols using biomaRt (version 2.42.0) in R and the org.Mm.eg.db (version 3.10.0) database. The contrasts fitted were between KO and WT damaged mice at 7 days and at 21 days. Significantly DE genes for GO and GSEA were defined as having a *p* value <0.05 and an absolute log_2_ FC >1 (Supplementary Data 5). The non-damage mice were not used in the differential expression calculation.

For the chronic damage model, comparison expressed genes were defined as having counts per million of at least 1 in half of the samples. Normalisation was done using calcNormFactors and variance estimators calculated using estimateDisp in edgeR. Differential expression analysis was performed using edgeR (version 3.18.1) and significantly DE genes were defined as having a FDR <10% and an absolute log_2_ FC >1 (Supplementary Data 1) while for comparison between chronic damage and partial hepatectomy DE genes were defined as either FDR < 0.1 or *p* value <0.05 and a log_2_ FC >1 depending on the number of significantly expressed genes (Supplementary Data 5).

#### Heatmaps, GO and GSEA

Heatmap analysis was performed by submitting RPKM values to the web tool ClustVis (https://biit.cs.ut.ee/clustvis/). GSEA was performed using the GSEA 4.0.3 software downloaded from http://software.broadinstitute.org/gsea/index.jsp. GO analysis was performed using the web tool DAVID (https://david.ncifcrf.gov).

#### IPA analysis

After statistical analysis, DE genes within groups were studied using the IPA (Qiagen). We imputed in IPA the whole transcriptome and then filtered for analysis focused on only statistically significant (*p* < 0.05) items with Log2CPM > 0.05 and −0.585 < Log_2_FC > 0.585. Pathways and “Upstream Regulators” showing relationships and interactions between DE genes and others that functionally interact with them, were generated and ranked in terms of the significance of participating genes (*p* < 0.05) and activation status (*Z*-score). A comparison analysis was then performed to focus only on those pathways significantly enriched. We considered “biologically relevant” only those genes that are statistically significant (*p* < 0.05), and enriched in “significantly modulated” pathways in the comparative analysis, and/or those genes with a FDR < 0.05. See details in Supplementary Data 2.

### Organoid cultures

#### Liver cholangiocyte organoids

Liver progenitor organoids were derived and cultured as previously described^41^. *Rnf43/Znrf3*^*del*^ organoids were generated by isolating cells from *Sox9CreERT-Rnf43/Znrf3*^*flox*^ mice and then treated in vitro with 3 μM 4-hydroxy-tamoxifen (Sigma) to induce deletion. For the isolation, livers were collected in PBS and dissociated using a collagenase solution (Collagenase type XI 0.012%, dispase 0.012%, FBS 1% in DMEM medium) and incubated for 3 to 4 h at 37 °C. The mixture was washed several times and ducts were hand-picked, mixed with Matrigel (BD Bioscience) and seeded in a 24 multi-well plate. After Matrigel had polymerised, the culture medium was added. Culture conditions for expansion (EM) were based on AdDMEM/F12 (ThermoFisher) supplemented with 1% B27 (Invitrogen), 1% N2 (Invitrogen), 1.25 mM N-acetylcysteine (Sigma-Aldrich), 10 nM gastrin (Sigma-Aldrich), 50 ng/ml mEGF (Peprotech), 5% RSPO1 conditioned medium (homemade), 100 ng/ml Fgf10 (Peprotech), 10 mM nicotinamide (Sigma-Aldrich) and 50 ng/ml HGF (Peprotech). To establish the culture, media was supplemented with 25 ng/ml Noggin (Peprotech), 30% Wnt3a conditioned medium (home-made) and 10 μm Rho-kinase inhibitor (Y27632, Sigma-Aldrich), for the first week. To generate *Rnf43/Znrf3*^*del*^ tumouroids from eHCC lesions, RSPO1 and WNT conditioned medium were removed. Weekly, organoids were removed from the matrigel, mechanically dissociated with a narrowed Pasteur pipette and transferred to fresh matrix in a 1:4 split ratio. Organoid pictures were taken using a scope and LAS V4.13 software (Leica).

To differentiate cultures into hepatocyte-like cells, liver progenitor organoids were cultured and differentiated as previously described^41^. Organoids were collected, disrupted by pipetting, seeded in matrigel and cultured in a complete expansion medium for 4 days. On day 5, medium was changed to differentiation medium (DM) based on AdDMEM/F12 (ThermoFisher) supplemented with 1% B27 (Invitrogen), 1% N2 (Invitrogen), 1.25 mM N-acetylcysteine (Sigma-Aldrich), 50 ng/ml mEGF (Peprotech), 50 nM A8301 (Tocris), 10 μM DAPT (Sigma) and 100 ng/ml FGF10 (Peprotech). The medium was refreshed every two days. Organoids were cultured in DM for a total of 9 days. Dexamethasone (3 μM) was added to the differentiation medium at the end of the protocol for a total of 3 days when organs were collected for analysis. When required, DM organoids were treated with 2 Wnt-inhibitors:  the porcupine inhibitor IWP2 (3 μM) and iCRT3 (25 μM) (Wnti medium).

#### Liver hepatocyte organoids

To obtain adult hepatocyte organoids, livers were perfused as described above. The resulting hepatocyte suspension was then filtered through a strainer (pore size of 100 μm) followed by centrifugation for 5 min at 50 × *g* and at 4 °C. After washing the obtained cell pellet with suspension buffer and centrifuging as before, the resulting pellet was resuspended in suspension buffer by gentle inversion for cell quantification and viability assessment. Five thousand viable hepatocytes were eventually plated in 20 μl matrigel and cultured in expansion media (Hepatocyte medium as described in^33^). R&Z^del^ hepatocytes were generated by either viral infection using AAV8-TBG-Cre particles (MOI = 100, 60 min at 37 °C) before cells were plated in matrigel for organoid culture establishment or treatment with 6 μM 4-hydroxy-tamoxifen (Sigma) for 48 h after plating. After deletion, cells were allowed to recover for 7 days in expansion media before splitting for experiments. For bodipy, R&Z^flox^ and R&Z^del^ organoids were grown in expansion media or in the presence of 2 Wnt-inhibitors: IWP2 (3 μM) and iCRT3 (25 μM) (Wnti medium), or 30% Wnt3a conditioned media (+Wnt3a) for 7 days before collection. For assessment of differentiation, R&Z^flox^ and R&Z^del^ organoids were grown either in expansion media or DM for 7 days before collection. Additionally, R&Z^flox^ organoids were also treated with Wnti in expansion media (with CHIR99021 and RSPO1) or 30% Wnt3a conditioned media in expansion media, or in expansion media without other WNT activators (no CHIR and no RSPO1) for 7 days before collection. Finally, 24 h before collection, media was changed and the eventual supernatant was collected for measuring albumin secretion using the Mouse Albumin AssayMax ELISA Kit (Assay Pro).

#### Hepatoblast and Human tumour organoids

Embryo hepatoblast organoids were obtained and cultured in hepatocyte medium as previously described^32^. To obtain R&Z^del^ organoids, after isolation, cells were treated with 3 μM 4-hydroxy-tamoxifen (Sigma) for 48 h and then washed and allowed to recover for an additional 7 days. After splitting, organoids were maintained in expansion media for 7 days before collection for bodipy and differentiation analysis. For differentiation experiments, cultures were supplemented with Wnt inhibitors as described in the hepatocyte organoid section above. Human tumouroids were cultured as previously described^43^ in AdDMEM/F12 (ThermoFisher) supplemented with 1% B27 (Invitrogen), 1% N2 (Invitrogen), 1.25 mM *N*-acetylcysteine (Sigma-Aldrich), 10 nM gastrin (Sigma-Aldrich), 50 ng/ml mEGF (Peprotech), 10% RSPO1 conditioned medium (home-made), 100 ng/ml Fgf10 (Peprotech), 10 mM nicotinamide (Sigma-Aldrich), 25 ng/ml HGF (Peprotech), 10 μM forskolin and 5 μM A8301.

### Organoid staining

Organoids were collected using a plastic Pasteur pipette, washed with cold PBS several times to remove matrigel or BME carefully to not disrupt their 3D structure. Subsequently, organoids were fixed for 40 min on ice with 10% Formalin. Fixed organoids were stored at 4 °C. For staining, fixed organoids were blocked with 1% bovine serum albumin (BSA), 2% donkey serum, 1% DMSO in PBS for 2 h at RT. Primary antibody was incubated o/n at 4 °C in 0.05% BSA in PBS. The secondary antibodies were added at a concentration of 1:150 for 2 h at RT. Nuclei were stained with Hoechst 33342 (Invitrogen) at a dilution 1:1000 for 15 min at RT. To assess lipid accumulation, cells were stained using BODIPY 493/503 dye (2 μM in PBS). BODIPY was added after the incubation with the membrane dye phalloidin. BODIPY was incubated for 3 h at RT protected from light. Imaging was performed using an SP8 White Light inverted confocal microscope or an Olympus Fluoview FV1000 confocal. The images were edited and analysed using the Leica Application Suite X software (LAS X, v1.5.1.1387) and ImageJ (v1.51j8).

### ZNRF3/RNF43 human mutation survival and expression analysis

To assess the effect of *RNF43/ZNRF3* mutations in human primary liver tumours we took advantage of the publicly available ICGC data collections, precisely, from Japan (LIRI-JP) and LICA-FR studies from the ICGC database^48,49^. We downloaded the RNAseq data for both studies. These data sets also included WGS of patients and corresponding survival data. Details on filtering methods and mutation and expression analysis can be found in Supplementary Methods.

#### Survival analysis

For the survival analysis donors were filtered to include those passing the quality control of the PCAWG^69^ study and containing associated tumour stage information. There were 257 donors in the LIRI-JP study and 1 in LICA-FR meeting the above-selected criteria. As the LICA-FR study only included 1 donor meeting our criteria the remaining survival analysis was performed using the LIRI-JP data set only.

We included a combined tumour stage covariate in the survival analysis based on the tumour stage at diagnosis information in the clinical data. Patients were split into two groups, those whose tumour stage was either 0 or 1 and those whose tumour stage was 2, 3 or 4. The sex of the donor was also used as a covariate in the model. As covariates, we also considered the effect of having a ZNRF3 and RNF43 mutation (ZandR). That left a sample size of 119 donors in total (no WNT mutations), 48 of these had ZNRF3 or RNF43 mutants with 38 having a mutation in ZNRF3 and 71 patients with no mutations (WT tumours). The number of patients with single RNF43 mutations or double mutation and fulfilling the criteria of no other mutations in the WNT pathway was too low to provide meaningful conclusions. To compare the double mutation to a single mutation in at least one of ZNRF3 or RNF43, we included in one model a separate term for each of ZNRF3 and RNF43 single mutations. In a second model, we included a combined ZNRF3 or RNF43 mutation factor (ZorR), in which a donor had at least one mutation in ZNRF3 or RNF43.

To test for differences in the effect of ZorR mutation on survival depending on the presence of Wnt mutations, we also fitted an interaction term between ZorR and Wnt.

The assumption of the proportional hazards used in the Cox regression model was tested using the Cox proportional hazards test (cox.zph) in the survival package in R. We accepted the null hypothesis of the proportional hazards model for all covariates except Wnt mutation status (*p* value <0.1) when using the ZorR covariate in the interaction model. Therefore, the data were split according to Wnt status.

For the donors with no mutation in Wnt, we fitted two multiple cox regression models using factors ZandR, sex and the combined tumour stage factor in each. We then included either two factors one for each ZNRF3 or RNF43 mutation in one model (Single model). In the second model, we included a combined OR factor for ZNRF3/RNF43 mutation status that was 1 if the patient had a mutation in at least one of ZNRF3 or RNF43, 0 otherwise (Combined model).

In the Wnt WT model, there were 15 with RNF43, 38 with ZNRF3 mutations, 48 with a mutation in RNF43 or RNF43 and 5 with a mutation in both. In the Wnt mutant group, there were 138 patients in total, 25 with double mutation and 54 with ZNRF3 and 32 with RNF43 mutation. Differences between the survival curves were tested using the Wald statistic and Kaplan–Meier curves plotted using the survival (version 3.2-7) and GGally (version 2.1.1) packages in R.

#### Gene expression analysis

For the differential gene expression analysis, we used RNAseq samples from the LIRI-JP and LICA-FR data sets. We included patients who also had corresponding mutation data from WGS, excluding those where WES was used. In total, 282 donors had RNAseq data with matching WGS available with 240 from LIRI-JP and 42 from LICA-FR. Genes were filtered to remove those not expressed, where expressed genes are defined as having counts per million >1 in >150 samples across the two data sets. Mutation status was determined as detailed above.

A multiple regression model was fitted in R using edgeR. The regression model contained four terms, one for each ZNRF3 OR RNF43 mutation status, ZNRF3 AND RNF43 mutation status, WNT mutation status and the fourth for the batch of origin of the sample LIRI-JP or LICA-FR. The normalisation of RNAseq samples was done using calcNormFactors and variance estimates calculated using estimateDisp in edgeR.

Differential gene expression analysis was calculated between WT and ZNRF3 OR RNF43 patients (having a mutation in at least one of ZNRF3 or RNF43, RorZ), between WT and double ZNRF3/RNF43 mutation (R&Z) and finally between WT and WNT mutation (WNT). There were 67 WT donors and 123 RorZ patients; 33 patients had a mutation in both R&Z and 177 patients had a mutation in one of the WNT genes. Genes were defined as DE between conditions if the FDR was <10%. GSEA against a defined set of lipid metabolism gene set (Supplementary Data 6_S24) was performed using the GSEA 3.0 software downloaded from http://software.broadinstitute.org/gsea/index.jsp.

For TCF4 enrichment in human gene expression data, we ranked the differential expression of three factors (Wnt mutation status, ZNRF3 or RNF43 mutation status and ZNRF3 and RNF43 mutation status) versus WT from the multiple regression model. A set of genes containing a TCF4 binding site within 5 kb (189 genes) was used to perform GSEA against a ranked list of DE genes in both models (vs all (left) and vs non-WNT mutants (right)). The top (first ranked) position had the gene with the highest increase in expression compared to the WT for each group. The ranked list was compared to the set of TCF4 genes (Supplementary Data 6_S45) and the maximum absolute enrichment score was calculated for each gene set. A positive enrichment score indicative of enrichment for TCF4 genes was upregulated (increase in expression) when compared to the WT.

For the comparison between the gene expression of our mutant mice and the human gene expression of HCC patients mutated in RNF43 and/or ZNRF3, GSEA was performed using genes DE between human patients with ZNRF3 and/or RNF43 mutations compared to non-WNT mutant patients and human patients with WNT vs non-WNT mutant patients regardless of their ZNRF3/RNF43 mutation status.

### Statistical analysis

All statistical methods are reported in the figure legends. Panels where several data points were quantified per mouse were plotted as a mean showing the distribution of all data points and analysed using a *t* test of means. When required, the data were tested and confirmed to be normally distributed by performing a normality test (Shapiro–Wilk test).

### Reporting summary

Further information on research design is available in the Nature Research Reporting Summary linked to this article.

## Supplementary information

Supplementary Information

Peer Review File

Description of Additional Supplementary Files

Dataset 1

Dataset 2

Dataset 3

Dataset 4

dataset 5

Dataset 6

Dataset 7

Reporting Summary

## Data Availability

All mouse gene expression data generated in this study have been deposited in the Gene Expression Omnibus (GEO) under accession code GSE133213. LIPID MAPS® structure was obtained from the owner’s webpage [https://www.lipidmaps.org/data/structure/]. TCF4 targets were determined using a publicly available data set [https://www.ncbi.nlm.nih.gov/geo/query/acc.cgi?acc=GSE41284]. LICA-FR and LIRI-JP data sets were obtained from the ICGC database [https://daco.icgc.org/]. Source data are provided with this paper.

## Source data

Source Data
