# Supplementary material for: RNF43/ZNRF3 loss predisposes to hepatocellular-carcinoma by impairing liver regeneration and altering the liver lipid metabolic ground-state
Source: Nat Commun. 2022 Jan 17;13:334. doi: 10.1038/s41467-021-27923-z (PMC8764073; doi:10.1038/s41467-021-27923-z)
Supplement: Supplementary file 1 — Supplementary Information [file 41467_2021_27923_MOESM1_ESM.pdf]

Supplementary Information

RNF43/ZNRF3 loss predisposes to Hepatocellular-carcinoma by impairing liver regeneration and altering the lipid metabolic ground-state

- Supplementary Figures 1-8
- Supplementary Table 1
- Supplementary Methods
- Supplementary References

Supplementary figure 1

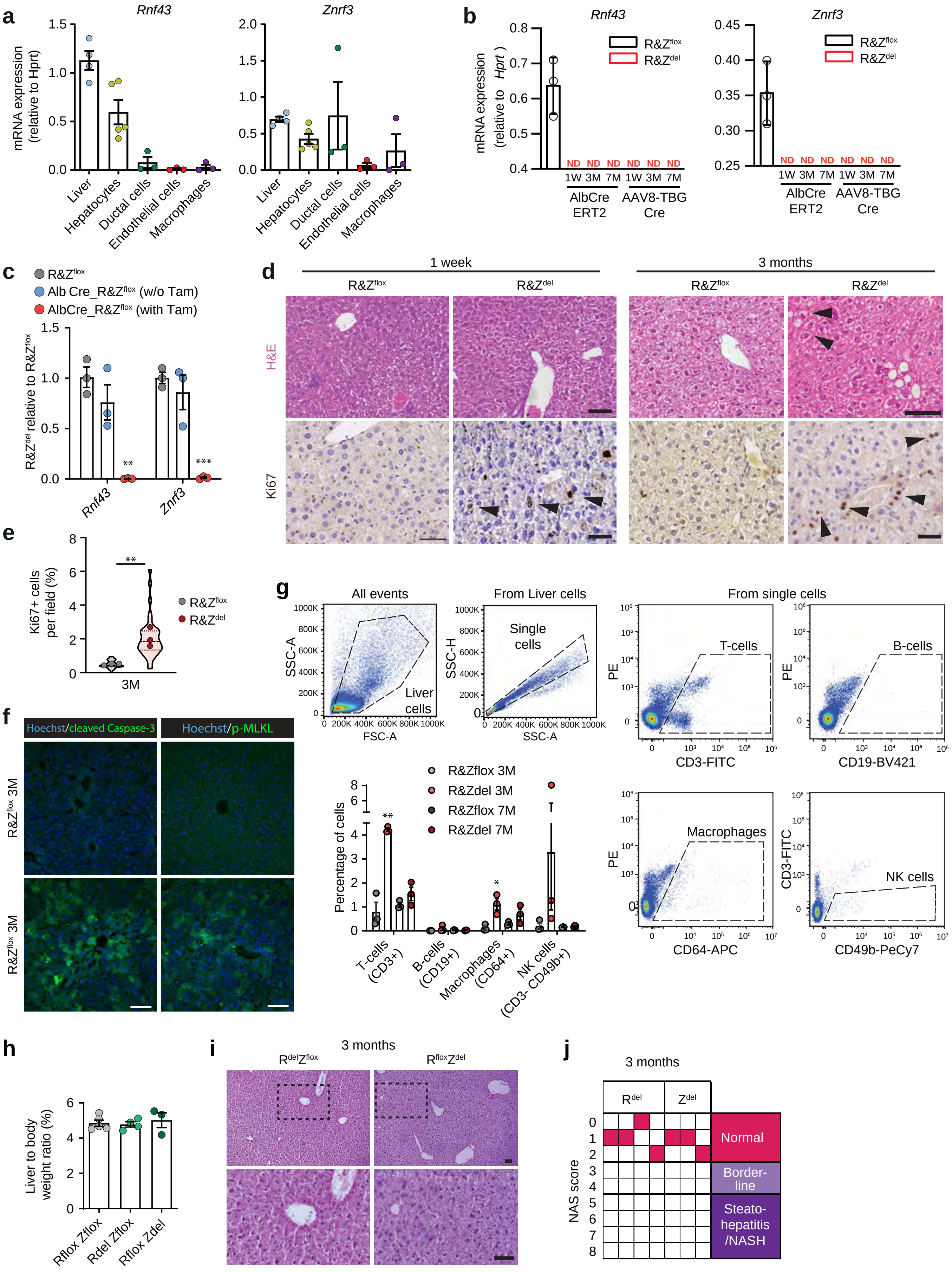

Supplementary Fig. 1. Liver-specific *Rnf43 & Znrf3* (R&Z) deletion induces hepatocyte proliferation, hepatomegaly, tissue degeneration and steatohepatitis. a qRT-PCR expression analysis of *Rnf43*/*Znrf3* in total liver (n=4), hepatocytes (n=5), ductal cells (EpCAM+, n=3), endothelial cells (CD31+, n=3) and macrophages (CD11b+, n=3). Graph represents mean +/- SEM. **b-c** Analysis of *Rnf43*/*Znrf3* gene deletion using qPCR primers that amplify the deleted exonic region. Results are expressed as mRNA expression relative to *Hprt*. **b** Analysis of *Rnf43*/*Znrf3* gene deletion in tamoxifen-injected *AlbCreERT2-R&Z^{del}* and AAV8-Cre-injected R&Z^{del} mice and non-induced R&Z^{flox}. Data represent mean ± SD, n=3. 1W, 1 week; 3M, 3 months; 7M, 7 months; *nd*, not detected. **c** Analysis of *Rnf43*/*Znrf3* deletion in *AlbCreERT2-R&Z^{flox}* 7M old mice injected with tamoxifen or vehicle. Data is represented as mean +/- SEM, n=3. Two-way ANOVA with Tukey multiple comparison; *Rnf43*, ***p*=0.001; *Znrf3*, ****p*=0.0004. **d-e** Histopathological analysis of R&Z^{flox} and R&Z^{del} livers at 1 week or 3 months post-deletion (3M). **d** Representative H&E and Ki67 immunostainings. Scale bar, 100µm (H&E) and 50µm (Ki67). Arrowhead, ballooned hepatocytes (top) and Ki67+ cells (bottom). **e** Graphs represent the number of Ki67+ hepatocytes per field-of-view (FOV, n=10). Violin plot represents median, IQR and full distribution of all FOVs. Dot, mean of FOV per mouse (n=3). Unpaired two-tail t-test of means, ***p*=0.0062. **f** Cleaved Caspase-3 (apoptosis) and p-MLKL (necroptosis) analysis in control and R&Z^{del} livers at 3M. Representative images (n=3 mice). Scale bar, 100 µm. **g** Flow cytometry analysis of immune cell populations in R&Z^{flox} and R&Z^{del}. Note the increase in T-cells (CD3+) and macrophages (CD64+) in R&Z^{del} livers. Graphs represent mean +/- SEM of n=3 mice. Unpaired two-tail t-test; T-cell, ***p*=0.001; Macrophages, **p*=0.0165. **h** Mean +/- SEM % of liver-to-body weight ratio of wild-type (R&Z^{flox} n=5) or single mutants R^{del}Z^{flox} (n=4) and R^{flox}Z^{del} (n=3). **i** Histopathological analysis (H&E) of R^{del}Z^{flox} and R^{flox}Z^{del} livers at 3 months post-deletion. Representative pictures (n=3 mice). Scale bar, 100µm. **j** NAS score of R^{del}&Z^{flox} and R^{flox}&Z^{del} single mutant mice (1 mice/ column). Pink =NAS 0-2= Normal; clear violet =NAS 3-4= borderline; dark violet =NAS 5-8= steatohepatitis/NASH. Source data in Source Data file.

Supplementary figure 2

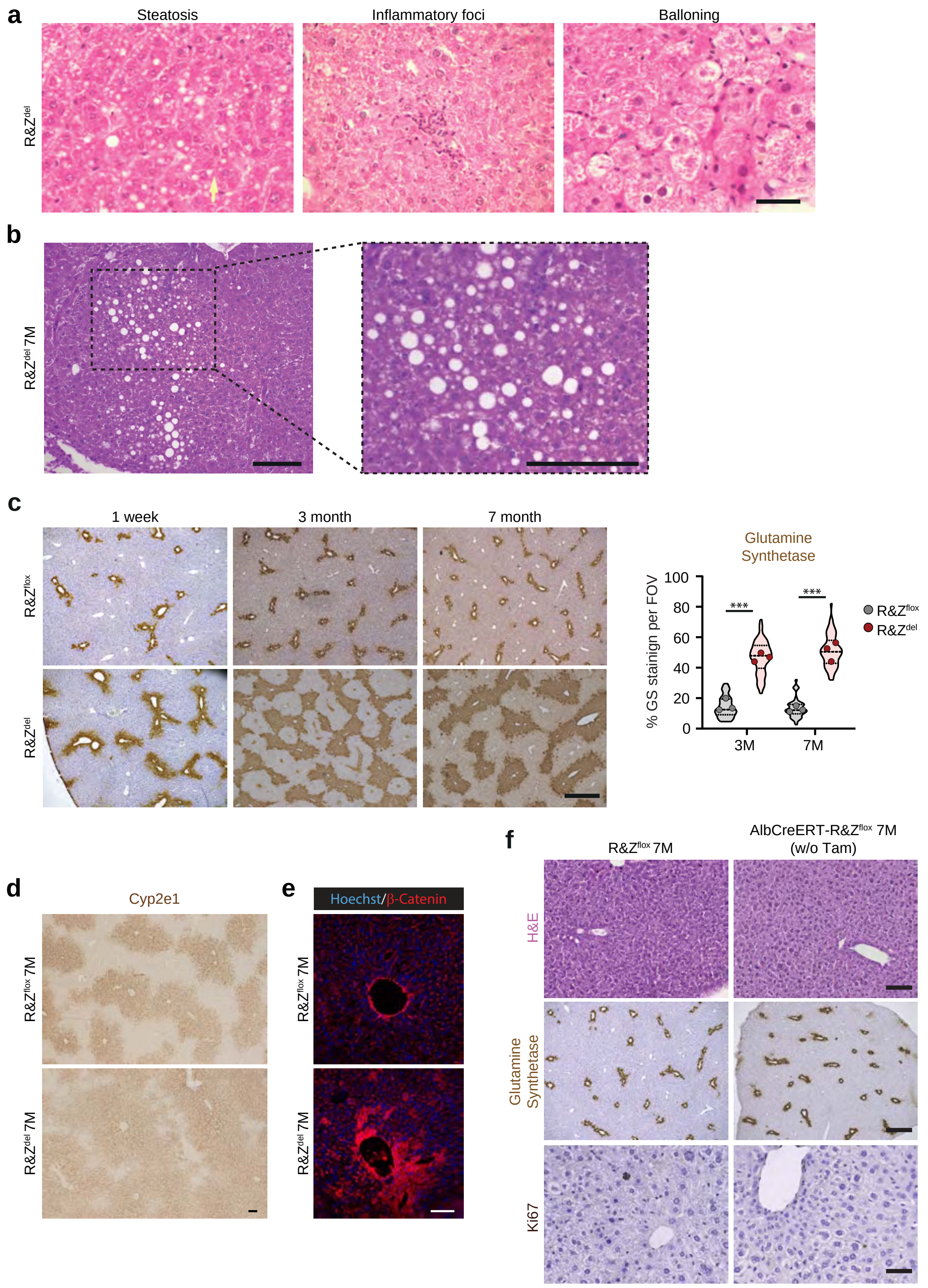

Supplementary Fig. 2. R&Z^{del} mutant mice show dysregulation of Wnt canonical pathway and features of steatohepatitis/NASH. a The presence of a steatohepatitis/NASH in R&Z^{del} livers was assessed by NAS score. Representative images of *Rnf43/Znrf3^{flox}* mice showing the 3 histological features used to categorize the samples: steatosis, inflammatory foci and cell ballooning. Scale bar, 50µm. **b** Representative pictures of benign microadenomas found in 10% (2 independent samples) of R&Z^{del} livers at 7 months after deletion. Scale bar, 100µm. **c** Representative pictures of GS staining in R&Z^{flox} and R&Z^{del} livers at the indicated time points after deletion. Scale bar, 500µm. Graph represents the quantification of GS (Glutamine Synthetase) area. Data is represented as violin plot showing median, IQR and full distribution of all FOVs (10-20 per mouse) counted. Each dot represents the mean of an independent mouse, n=3 mice per group. Two-way ANOVA with Sidak multiple comparison. 3M and 7M, ***p=0.0001. **d** CYP2E1 immunohistochemistry analysis in wild-type and R&Z knock-out liver sections at 7 months post deletion. 3 mice per group were analyzed. Scale bar, 100 µm. **e** β-Catenin immunofluorescence staining in R&Z^{flox} and R&Z^{del} liver sections at 7 months post deletion. Representative images of 3 mice per group are shown. A higher Wnt activity around liver central veins is observed in mutant mice. Scale bar, 100 µm. **f** Histopathological analysis of R&Z^{flox} and of *Alb*CreERT-R&Z^{flox} mice not treated with tamoxifen (w/o Tam) at 7 months after deletion. Representative pictures of at least 3 independent biological replicates are shown. Scale bar, 100µm (H&E), 500µm (GS) and 50µm (Ki67). Source data are provided as a Source Data file.

Supplementary figure 3

a

Lipid metabolism · Cancer · Inflammation and cell damage

Diseases and Bio Functions

	1M	3M	7M
Synthesis of sterol	N/A	2.502	1.408
Conversion of lipid	0.511	2.416	1.42
Conversion of fatty acid	N/A	2.2	1.671
Conjugation of glutathione	N/A	2.192	1.951
Liver cancer	0.412	2.187	1.636
Metabolism of cholesterol	0.204	2.106	1.757
Synthesis of cholesterol	N/A	2.106	1.481
Uptake of lipid	N/A	2.037	1.512
Transport of steroid	N/A	2.028	1.57
Gastrointestinal adenocarcinoma	N/A	1.939	1
Intestinal adenocarcinoma	N/A	1.939	1
Hepatobiliary system cancer	-0.152	1.866	1.636
Hepatocellular carcinoma	-0.396	1.834	1.672
Liver carcinoma	N/A	1.834	1.25
Transport of lipid	N/A	1.785	1.213
Digestive system cancer	-0.82	1.717	1.011
Gluconeogenesis	N/A	1.709	1.746
Metabolism of sterol	0.182	1.701	1.64
Synthesis of terpenoid	N/A	1.496	1.875
Intestinal carcinoma	-1.091	1.475	1
Hepatobiliary carcinoma	-0.976	1.465	1.25
Synthesis of steroid	0.053	1.461	1.779
Gastrointestinal tumor	-0.615	1.4	1.324
Metabolism of terpenoid	0.625	1.331	2.994
Metabolism of xenobiotic	N/A	1.292	1.947
Intestinal tumor	-1.075	1.257	1.449
Hydroxylation of lipid	N/A	1.24	1.284
Cell viability	-0.616	1.114	1.537
Cell survival	-0.454	1.112	1.663
Oxidation of lipid	N/A	1.064	2.362
Steroid metabolism	0.272	1.005	2.488
Transport of molecule	1.453	2.107	0.947
Glucose metabolism disorder	1.038	1.666	0.049
Cell death of epithelial cells	2.203	1.45	-1.087
Necrosis of epithelial tissue	2.301	1.417	-0.904
Inflammation of absolute anatomical region	1.421	0.674	1.277
Inflammation of organ	1.014	0.289	1.199

R&Z^{del} vs R&Z^{flox}

b

Upstream Regulators

	1M	3M	7M
LGR4	4.08	3.89	4.31
LGR5	3.98	4.1	4.22
EIF4E	2.34	2.14	3.05
CTNNB1	1.61	3.54	3.05
TCF7L2	1.31	3.02	3.03
INSIG1	-1.96	-3.49	-2.76
TXNRD1	-2.24	-2.14	-2
GSR	-2.24	-1.9	-2
ACOX1	-2.3	-1.78	-1.53
CDK19	-2	-2.71	-1.49

c

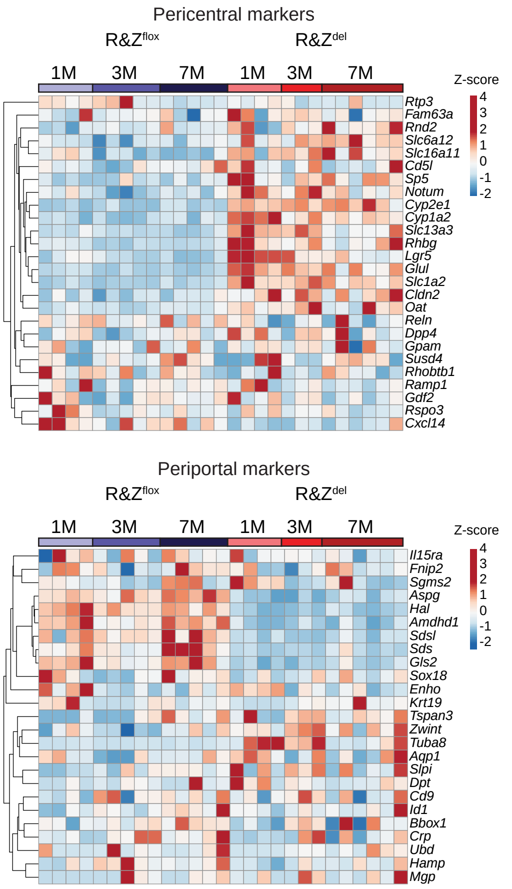

d

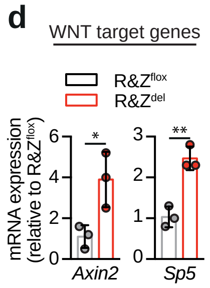

e

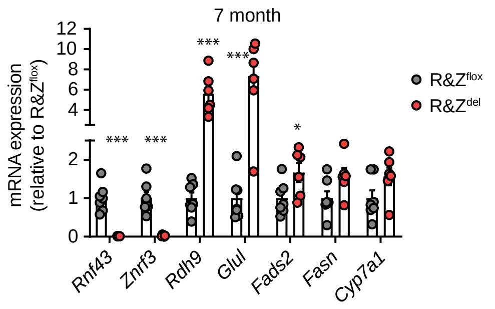

Supplementary Fig. 3. Constitutive activation of the WNT canonical pathway in *Rnf43 & Znrf3* (R&Z) null hepatocytes results in lipid metabolic changes. a-b Ingenuity Pathway Analysis (IPA) categorization of genes up-regulated in *Rnf43 & Znrf3* mutant (R&Z^{del}) compared to WT (R&Z^{flox}) livers at different time-points. Positive values (red) mean enrichment while negative values (blue) mean decreased expression levels. Number indicates activation Z-score. **a** Disease pathways and bio functions related to lipid metabolism, cancer and inflammation as identified by IPA analysis. **b** Top upstream regulators of R&Z^{del} transcriptome as identified by IPA analysis. **c** Heat-map analysis of the RPKM values (raw z-scored) of liver pericentral (top) and periportal (bottom) genes indicating that liver metabolic zonation is disrupted in *Rnf43/Znrf3* mutant livers. **d** Expression analysis (q-RT-PCR) of the indicated Wnt target genes. Graph represents mean±SD of n=3 mice. Unpaired two tail t-test; *Axin2,* **p*=0.0295; *Sp5,* ***p*=0.0029. **e** qRT-PCR expression analysis for *Rnf43* and *Znrf3* and lipid metabolic genes in mutant and non-induced littermates at 7 months after deletion. Graph represents mean±SEM, n=7 mice in R&Z^{flox} group, n=6 mice in R&Z^{del} group. Unpaired two-tail t-test; *Rnf43,* ****p*=0.00005; *Znrf3,* ****p*=0.0001; *Rdh9,* ****p*=0.0001; *Glul,* ****p*=0.0003; *Fads2*, **p*=0.038. Source data are provided as a Source Data file.

Supplementary figure 4

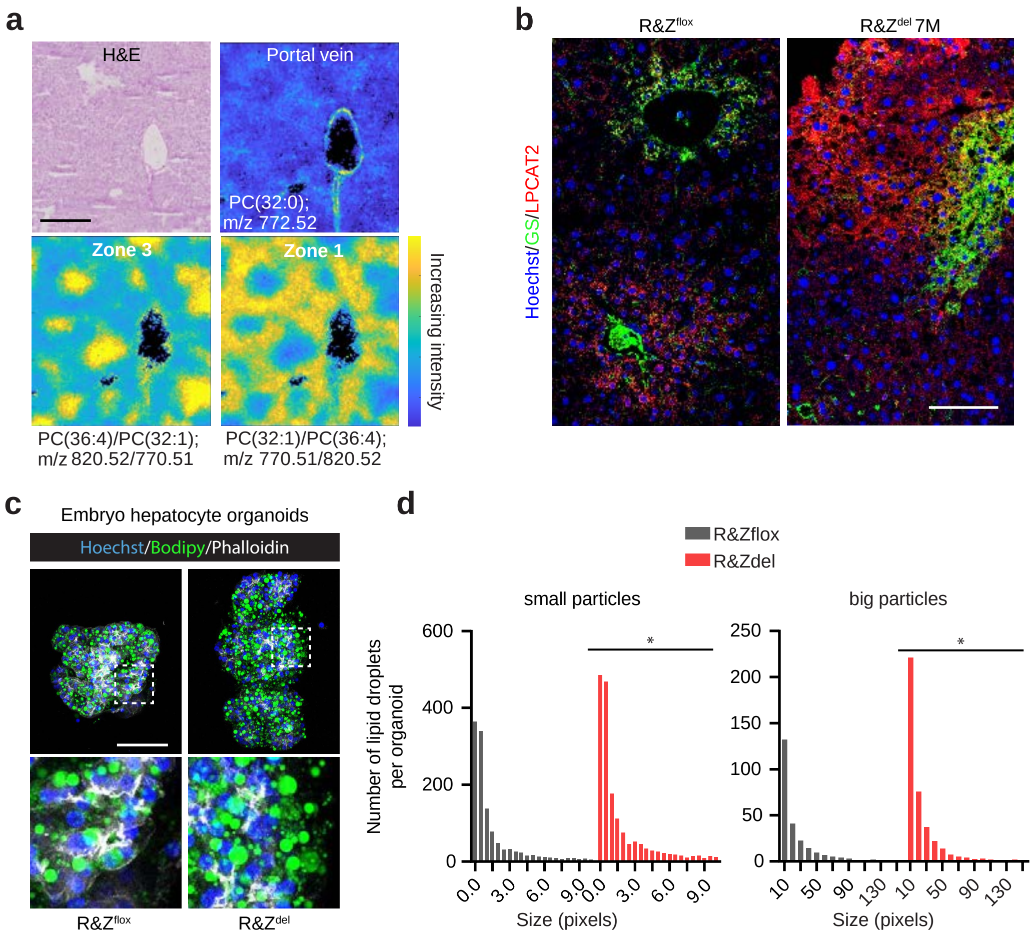

Supplementary Fig. 4. *Rnf43/ Znrf3* null hepatocytes show aberrant lipid zonation and a cell-autonomous accumulation of lipid droplets. a MS imaging shows the spatial distribution of key lipids (or ratios of lipids) which closely recapitulate periportal (zone 1) and perivenous/pericentral (zone 3) regions. Representative images of 3 independent samples are shown. Scale bar, 500 μM. **b** Immunostaining analysis for the portal vein marker LPCAT2 and central vein maker Glutamine synthetase (GS). Note that the expression of LPCAT2 in *Rnf43/Znrf3* mutant livers partially overlaps with the GS+ central vein area. Representative images of 2 different mice per group are shown. Scale bar, 100 μm. **c-d** Mouse hepatocyte organoids derived from *Alb*CreERT-R&Z^{flox} embryos were obtained, treated with hydroxy-tamoxifen in vitro to induce R&Z deletion and then analysed for their lipid droplet content using Bodipy staining (neutral lipids). **c** Representative images are shown. Scale bar, 100 μm. **d** Quantification of the number of small (0-9 pixel size) and big (10-150 pixel size) lipid droplets present in R&Z^{flox} and R&Z^{del} cells from embryonic hepatocyte organoids. R&Z^{flox} n=3, R&Z^{del} n=4. Two-way ANOVA with Tukey's multiple comparison; Small particles, *p=0.0133; Big particles, *p=0.0215. Source data are provided as a Source Data file.

Supplementary figure 5

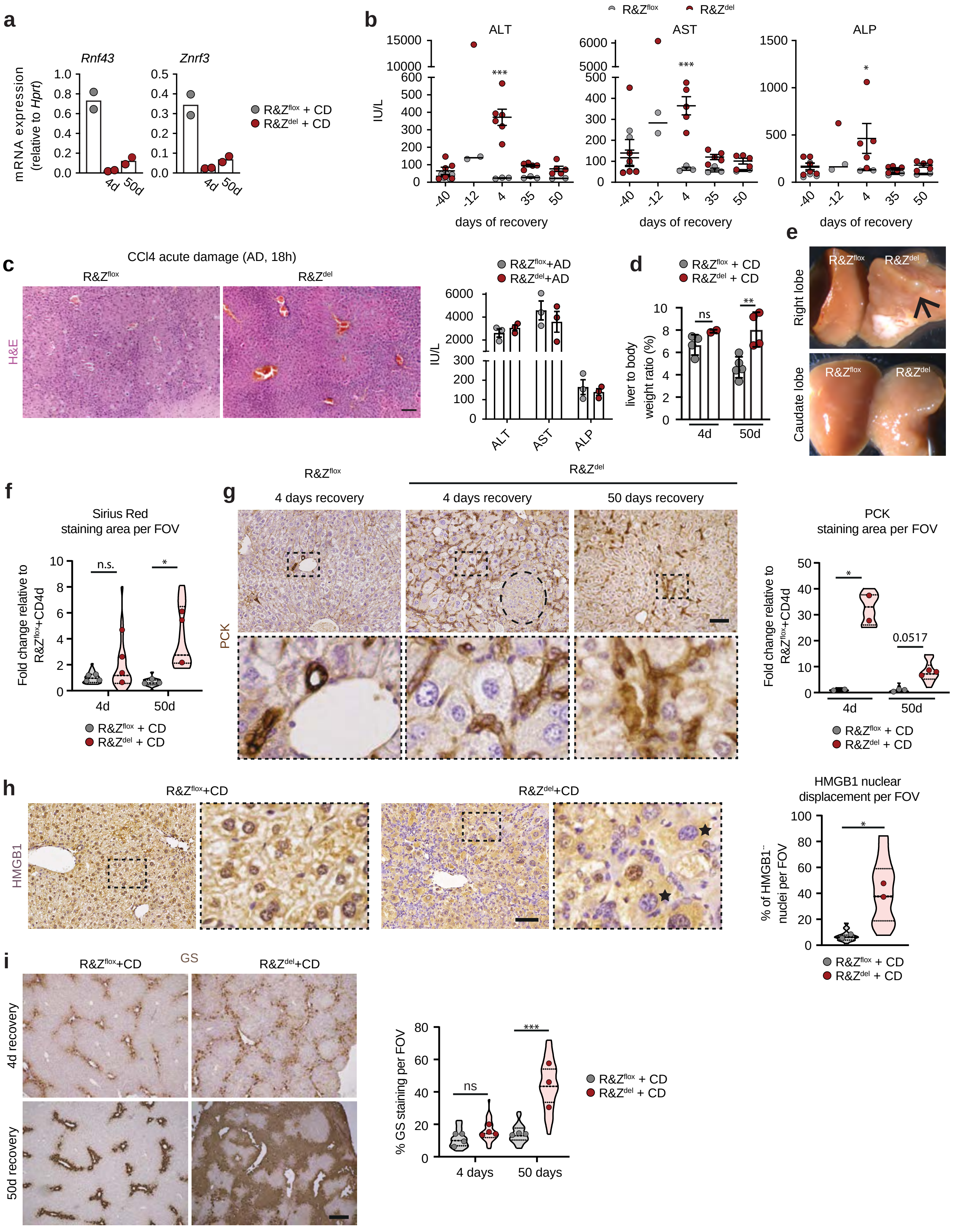

Supplementary Fig. 5. *Rnf43/Znrf3* mutant livers exhibit impaired regeneration upon CCl_4-chronic liver damage. a Analysis of *Rnf43*/*Znrf3* gene deletion in R&Z^{flox} and R&Z^{del} mice at 4 and 50 days recovery after CCl4 chronic damage (CD). Results are expressed as mRNA expression relative to *Hprt*. Data represent mean ± SD, n=2. **b** ALT, AST and ALP levels. Analysis was performed at -40 days (before damage), -12 days (during damage) and 4, 34 and 50 days post-damage (recovery). R&Z^{flox}, n=3 (time 0, 4, 34 and 50 and n=2 time -12); R&Z^{del}, n=3 (time 0), n=2 (time -12) and n=5 (time 4, 34 and 50). Two-way ANOVA and post-hoc Sidak test; ALT, ***p=0.0001; AST, ***p=0.0003; ALP, p=0.0222. **c** Control and R&Z^{del} mice received an acute dose (AD, see methods) of CCl_4 and serum and livers were collected 18h later. Representative H&E stainings. Scale bar, 100 µm. Graphs represents the mean +/- SEM of the AST, ALT and ALP levels, n=3. **d** Mean +/- SD of the % of liver-to-body weight ratio of R&Z^{flox} or R&Z^{del} mice. R&Z^{del}-4d, n=2; R&Z^{flox}-50d, n=5; R&Z^{flox}-4d and R&Z^{del}-50d, n=4. Unpaired two-tail t-test. **p=0.0047. **e** Stereoscope pictures of R&Z^{flox} and R&Z^{del} livers at day 50 days of recovery. Arrow, irregular/nodular surface. **f** Graphs represent fold change increase of Pico-Sirius red staining area (see Figure 4) per field of view (FOV). Data is represented as violin plot showing median, IQR and full distribution of all FOVs (n=10/ mouse). Dot, mean of FOV per mice, (n=3-4 mice). Unpaired two-tail t-test of means. *p=0.0116. **g** Representative pictures of R&Z^{del} livers stained for PCK (pan-cytokeratin, ductular reaction). Dashed circle, regenerative nodule. Scale bar, 100µm. Graphs represent the fold change in PCK staining area relative to WT from the 4d recovery time point. Data is represented as violin plot showing median, IQR and full distribution of all FOVs (n=10). Dot, mean of FOV/mouse. Two-way ANOVA and post-hoc Sidak test; ***p=0.0001. **h** Representative images of HMGB1 cytoplasmic staining (cellular damage). Star, HMGB1 negative nuclei. Scale bar, 100µm. Graph shows quantification of HMGB1 nuclear displacement after 4 days of recovery. Violin plot represents the distribution of all FOV (n=10 per mouse) showing median and IQR. Dot, mean of FOV/mouse, n=3. Unpaired two-tail t-test, *p=0.0223. **i** Representative images of Glutamine Synthetase (GS) immunostaining after chronic damage. Scale bar, 500µm. Graph represents the GS staining area per FOV. Data is presented as violin plot showing median, IQR and full distribution of FOVs (n=10 per mouse). Dot, mean of FOV/mouse, n=3. Two-way ANOVA and post-hoc Sidak test; ***p=0.0004. Source data in Source Data file.

Supplementary figure 6

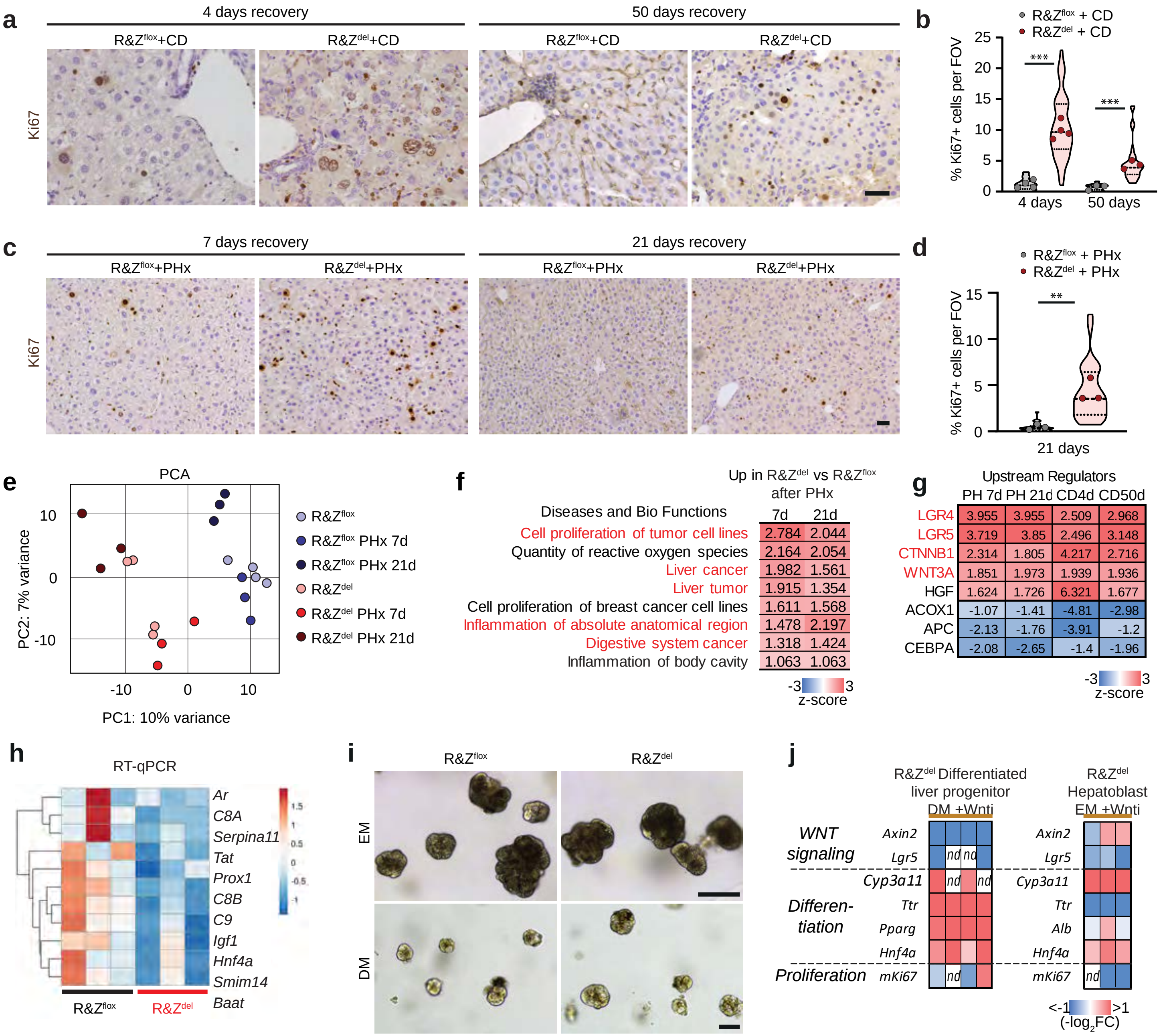

Supplementary Fig. 6. *Rnf43/Znrf3* deleted livers present excessive proliferation and impaired hepatocyte maturation following PHx and CCl_4-induced chronic damage. a-b R&Z^{del} livers show increased proliferative activity (Ki67+ cells) after CCl_4 chronic damage. **a** Representative images. Scale bar, 50µm. **b** Quantification of the number of Ki67+ cells per FOV. Violin plot represents median, IQR and full distribution of all FOVs counted (n= 10 FOV/mouse). Dot, mean of FOV/mouse, n=3-4 mice. Two-way ANOVA and post-hoc Sidak test; CD 4d, ***p=0.0001; CD 50d, ***p=0.0009. **c-d** After partial hepatectomy (PHx), R&Z^{del} livers show increased proliferative activity (Ki67+ cells). **c** Representative images. Scale bar, 500µm. **d** Quantification of the number of Ki67+ cells per FOV. Data is presented as violin plot showing median and IQR of n=3 mice per group (n=10 FOV/mice). Dots, independent mouse. Unpaired two-tail t-test of means was used. **p=0.0079. **e** PCA analysis showing wide transcriptomic differences between control and R&Z^{del} livers after PHx. **f-g** IPA categorization of genes up-regulated in R&Z^{del} compared to WT (R&Z^{flox}) livers after damage (PHx, **f-g**; Chronic damage-CD, **g**) at the indicated time points of recovery. Red, Upregulated. Blue, downregulated. Number, activation Z-score. **f** Common upregulated bio function pathways. **g** Common top upstream regulators associated to R&Z^{del} damaged transcriptome in both PHx and CCl_4 chronic damage (CD). Positive values (red), activated regulators. Negative values (blue), inhibited regulators. **h** qRTPCR analysis validation of the results in Figure 5f. Data are presented as a heat-map of the fold change relative to *Hprt* (raw z-scored) (right). **i** Brightfield pictures of R&Z^{flox} and R&Z^{del} adult hepatocyte organoids in expansion (EM) or differentiation (DM) medium. N=3 independent experiments. Scale bar, 100µm. **j** qPCR expression analysis of the indicated genes in R&Z^{del} differentiated liver progenitor or hepatoblast organoids grown in presence or not of Wnt-inhibitors. Heatmap represent the fold-change values of each biological replicate normalized to the respective R&Z^{flox} untreated control. Source data in Source Data file.

Supplementary figure 7

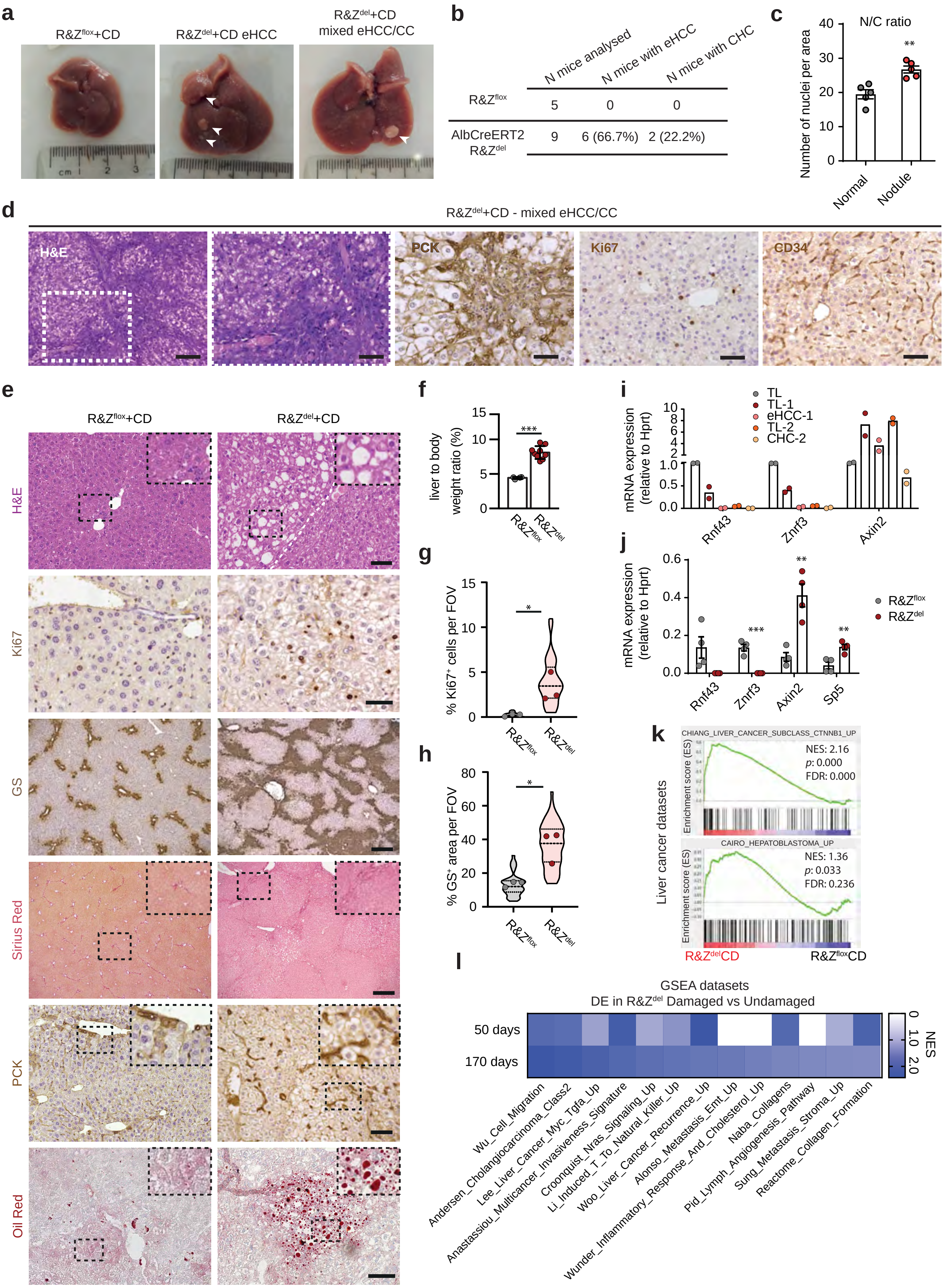

Supplementary Fig. 7. *Rnf43/Znrf3* deletion results in hepatocellular carcinoma and mixed HCC/CC tumours in mice. a-l *Rnf43/Znrf3* liver specific mutant mice were chronically damage (CD) by repetitive CCl_4 injections as described in Figure 6. Tissues were collected and analysed at 170 days of recovery(**a-l**) or 50 days recovery (**l**). **a** Stereoscope pictures of R&Z^{flox}+CD and R&Z^{del}+CD livers. **b** Penetrance of eHCC and CHC. **c** Mean +/- SEM of the nucleus-to-cytoplasm ratio in tumor nodules (defined by Collagen IV staining, see Figure 6) compared to the non-tumour adjacent tissue in R&Z^{del} livers (n=5). Paired two tail t-test, ***p*=0.0076. **d** Representative pictures of H&E staining, PCK, Ki67 and CD34 immunostaining of a mixed subtype neoplastic lesion (CHC). Scale bar, 100µm (H&E left panel); 50µm (PCK, Ki67, CD34 and H&E right panel). **e** Representative H&E, Ki67, GS, Sirius red, PCK and Oil Red O staining images. Scale bar, 200µm (H&E, GS, Sirius Red); 100µm (Oil red, PCK), 50µm (Ki67). White dashed line, nodule. **f** Graph showing the percentage of liver-to-body weight ratio in n=3 (R&Z^{flox}) and n=9 (R&Z^{del}) mice. Data represent mean ± SD. Unpaired two-tail t-test. ****p*=0.0001 **g-h** Quantification of Ki67 positive hepatocytes per FOV **(g)** and of GS positive staining localized around central veins **(h)**. Data represents median, IQR and distribution of FOV (n=10/mouse). Dot, mean of FOV/mice, n=3 mice. Unpaired two-tail t-test of means. Ki67 **p*=0.0422; GS **p*=0.0145. **i** Expression pattern (qRT-PCR) of *Rnf43, Znrf3 and Axin2* in eHCC and CHC lesions. Data represent mean, n=2. TL, total liver. eHCC or CHC, microdissected lesion. **j** qPCR expression analysis for WNT target genes in organoids from R&Z^{flox} and R&Z^{del} eHCC. Data represent mean +/- SEM, n=4. Unpaired two-tail t-test; *Znrf3*, ****p*=0.0002; *Axin2*, ***p*=0.0023; *Sp5*, ***p*=0.0053. **k** Plots showing normal enrichment score (NES) of selected liver cancer GSEA datasets significantly enriched in R&Z^{del} damaged livers (FDR<25%, pV<0.05, two-sided permutation test). **l** Heatmap showing normal enrichment score (NES) for selected GSEA datasets R&Z^{del} damaged livers against R&Z^{del} undamaged livers at 50 and 170 days of recovery. Two-sided permutation test; Blue, *p*<0.05. White, *p*>0.05. Source data in Source Data file.

Supplementary Figure 8

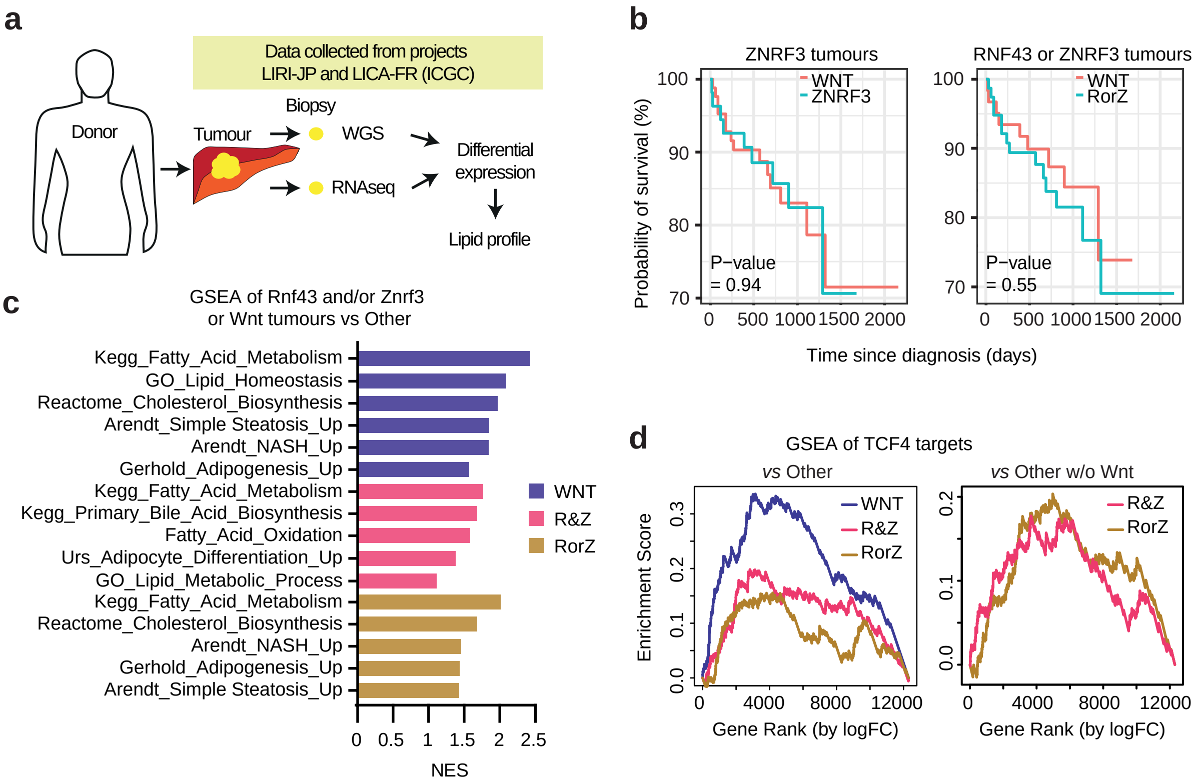

Supplementary Fig. 8. Human HCC patients mutated in RNF43 and/or ZNRF3 present lipid metabolic alterations and poor prognosis. a-d a Experimental design. Prognosis and expression pattern of human tumours mutated in *RNF43* and/or *ZNRF3* or in other WNT pathway components (*APC, AXIN1 or CTNNB1).* See also methods, Figure 7 and Supplementary Data 6. **b** Survival analysis after Cox proportional hazards model. Graph shows the survival analysis of HCC patients with ZNRF3 mutations (ZNRF3, left) or with mutations in RNF43 or ZNRF3 (RorZ, right) compared to patients with WNT mutations (WNT). Comparative to Figure 7 evidences the WNT-RNF43/ZNRF3 interaction. *p*-Values obtained by Wald statistic are shown, CI 95%. **c** Gene expression analysis of HCC patients with mutations in RNF43 and ZNRF3 (R&Z), RNF43 or ZNRF3 (RorZ) and mutations in other WNT pathway components (WNT). Graph shows significantly enriched (p-value<0.05) gene sets involved in lipid metabolism when the interaction between WNT mutation status in RNF43 and/or ZNRF3 patients is not taken into account. Compare to Figure 7d where the DE and GSEA takes into account the interaction. **d** TCF4 enrichment in human gene expression data was used as a proxy to determine whether the WNT pathway was active in the different patient groups. Left panel: Red, R&Z patients vs non-R&Z mutants regardless of mutations in WNT genes; Brown, RorZ mutant patients vs non-RorZ mutants regardless of mutations in WNT genes; Blue, WNT mutant patients vs non-WNT mutants. Right panel, GSEA plot for R&Z patients vs non-R&Z mutants excluding patients with mutations in WNT genes (Red); RorZ mutant patients vs non-RorZ mutants excluding patients with mutations in WNT (Brown). Results show positive enrichment for TCF4 target genes in both models, with higher enrichment for WNT compared to R&Z double knockout (*p*-value <0.001, two-sided permutation test) and higher score for double mutation compared to patients with at least one mutation in either RorZ (*p*-value 0.04, two-sided permutation test).

Supplementary Table 1. Cholesterol and Triglycerides blood levels.

Measure	Cholesterol (mmol/L)		Triglycerides (mmol/L)	
Genotype	R&Z^{flox}	R&Z^{del}	R&Z^{flox}	R&Z^{del}
mouse 1	4.21	3.35	3	1.2
mouse 2	4.58	3.56	2.3	1.5
mouse 3	5.01	2.57	1.4	1.8
mouse 4	2.15		1.3	1.2
MEAN	3.99	3.16	2.00	1.42
SD	1.27	0.52	0.80	0.29
p-value	0.3435		0.2267	

Supplementary Methods

Lipidomics analysis

Lipids were extracted from liver tissue using the Folch method. Briefly, liver tissue was homogenized in chloroform:methanol (2:1, 1 mL) using TissueLyzer (Qiagen Ltd., Manchester, UK). Deionized water (400 µL) was added, and the samples were well mixed. Separation of the aqueous and organic layers was carried out following centrifugation (12,000 *g*, 10 minutes). The lipid-containing organic extract was evaporated in a vacuum centrifuge, without heat, and stored at −80°C until analysis. Details on lipidomics analysis can be found in Supplementary Methods.

Lipid profiling was performed by liquid chromatography-mass spectrometry (LC-MS) using a Vanquish Flex Binary UHPLC system (Thermo Fisher Scientific) coupled to Orbitrap Q-Exactive mass spectrometer (Thermo Fisher Scientific). Chromatographic separation was achieved on Acquity UPLC BEH C18 column (Waters, 50 × 2.1 mm, 1.7 µm) at 55°C and flow rate of 0.5 mL/min. Mobile phase was composed of 60:40 (v/v) acetonitrile:water (solvent A) and 90:10 (v/v) isopropanol:acetonitrile (solvent B), each containing 10 mM ammonium formate (positive ion mode) or 10 mM ammonium acetate (negative ion mode). A gradient elution program was performed for both ion modes as described in[1]. Ionization was performed using a heated electrospray ionization source (HESI), with capillary voltage 3.5/-2.5 kV, heater temperature 438°C, capillary temperature 320°C, S-lens RF level 50, sheath, auxiliary and sweep gas flow rate are 53, 14 and 1 unit, respectively. High-resolution mass spectrometric (70,000 at *m/z* 200) data were acquired in profile mode (*m/z* 200-2000).

Lipidomics data acquisition was performed with Xcalibur software (version 4.1). Data were converted to mzML format and peak picking performed using XCMS[2], and features normalised to isotopically labelled internal standard of the same lipid class, and tissue weight. Orthogonal projection to latent structures discriminant analysis (OPLS-DA) models were constructed using SIMCA 14 (Umetrics, Sweden) with Pareto scaling. Volcano plot (combination of fold change and unpaired t-test P values for individual variables) were produced using Metaboanalyst[3] version 4.0. Heatmap was generated in Metaboanalyst using the Ward clustering method with Euclidean distance measure. Features were centered and scaled using the “Autoscale” function, and the top 20 hits based on their significance (ANOVA) were calculated. Lipid identification was performed by accurate mass using LIPID MAPS® Structure database[4] (< 5 ppm) (https://www.lipidmaps.org/data/structure/) and confirmed where possible with fragmentation by tandem mass spectrometry (LC-MS/MS).

RNA sequencing and analysis

RNA libraries were prepared for sequencing using the Smart-Seq2 protocol[5]. Details on sequencing and analysis can be found in Supplementary Methods.

RNA-Seq libraries were sequenced on an Illumina HiSeq 4000 instrument in single read mode at 50 base length. Reads were filtered for low quality (<Q20) with Sickle (version 1.33). Reads were then mapped to mm10 UCSC reference genome[6] using the STAR aligner (version 2.5.0a)[7] with the parameters "--outSAMmultNmax 1, --quantMode GeneCounts". Raw counts were generated using featureCounts (version 1.6.0)[8] software and includes all exons for a gene from the mm10 UCSC GTF file.

ZNRF3/RNF43 Human mutation survival and expression analysis

To assess the effect of *RNF43/ZNRF3* mutations in human primary liver tumours we took advantage of the publicly available ICGC data collections, precisely, from Japan (LIRI-JP) and LICA-FR studies from the ICGC database[9,10]. We downloaded the RNAseq data for both studies. These datasets also included WGS of patients and corresponding survival data. Details on filtering methods and mutation and expression analysis can be found in Supplementary Methods.

Mutation data was annotated and filtered based on their predicted consequence type under different metrics from SNP[11]. Somatic mutations were included if they met one of the following criteria: 1) SIFT prediction for coding variants was classed as deleterious with high confidence; 2) Polyphen prediction was damaging for coding variants; 3) Frameshift variants with stop gained as annotated by SNP nexus; 4) Eigen PC (non-coding) score was greater than zero; 5) FatHMM non-coding score was positive[12]; 6) Fit conservation score was greater than 0.2[13]; 7) DeepSEA p-value <0.1[14]; 8) FunSeq2 score was greater than 0.15[15]; 9) ReMM score greater than 0.5[16] and 10) CADD score of at least 6[17].

Patients harbouring mutations in either APC, AXIN1 or CTNNB1 were considered to have a mutation in WNT, regardless of their ZNRF3/RNF43 mutation status.

Supplementary References

1 Hall, Z. *et al.* Lipid Remodeling in Hepatocyte Proliferation and Hepatocellular Carcinoma. *Hepatology* **73**, 1028-1044, doi:10.1002/hep.31391 (2021).

2 Smith, C. A., Want, E. J., O'Maille, G., Abagyan, R. & Siuzdak, G. XCMS: processing mass spectrometry data for metabolite profiling using nonlinear peak alignment, matching, and identification. *Analytical chemistry* **78**, 779-787, doi:10.1021/ac051437y (2006).

3 Chong, J., Wishart, D. S. & Xia, J. Using MetaboAnalyst 4.0 for Comprehensive and Integrative Metabolomics Data Analysis. *Current protocols in bioinformatics* **68**, e86, doi:10.1002/cpbi.86 (2019).

4 Sud, M. *et al.* LMSD: LIPID MAPS structure database. *Nucleic acids research* **35**, D527-532, doi:10.1093/nar/gkl838 (2007).

5 Picelli, S. *et al.* Smart-seq2 for sensitive full-length transcriptome profiling in single cells. *Nature methods* **10**, 1096-1098, doi:10.1038/nmeth.2639 (2013).

6 Kent, W. J. *et al.* The human genome browser at UCSC. *Genome research* **12**, 996-1006, doi:10.1101/gr.229102 (2002).

7 Dobin, A. *et al.* STAR: ultrafast universal RNA-seq aligner. *Bioinformatics* **29**, 15-21, doi:10.1093/bioinformatics/bts635 (2013).

8 Liao, Y., Smyth, G. K. & Shi, W. featureCounts: an efficient general purpose program for assigning sequence reads to genomic features. *Bioinformatics* **30**, 923-930, doi:10.1093/bioinformatics/btt656 (2014).

9 International Cancer Genome, C. *et al.* International network of cancer genome projects. *Nature* **464**, 993-998, doi:10.1038/nature08987 (2010).

10 Zhang, J. *et al.* International Cancer Genome Consortium Data Portal--a one-stop shop for cancer genomics data. *Database : the journal of biological databases and curation* **2011**, bar026, doi:10.1093/database/bar026 (2011).

11 Dayem Ullah, A. Z. *et al.* SNPnexus: assessing the functional relevance of genetic variation to facilitate the promise of precision medicine. *Nucleic acids research* **46**, W109-W113, doi:10.1093/nar/gky399 (2018).

12 Shihab, H. A. *et al.* An integrative approach to predicting the functional effects of non-coding and coding sequence variation. *Bioinformatics* **31**, 1536-1543, doi:10.1093/bioinformatics/btv009 (2015).

13 Gulko, B., Hubisz, M. J., Gronau, I. & Siepel, A. A method for calculating probabilities of fitness consequences for point mutations across the human genome. *Nature genetics* **47**, 276-283, doi:10.1038/ng.3196 (2015).

14 Zhou, J. & Troyanskaya, O. G. Predicting effects of noncoding variants with deep learning-based sequence model. *Nature methods* **12**, 931-934, doi:10.1038/nmeth.3547 (2015).

15 Fu, Y. *et al.* FunSeq2: a framework for prioritizing noncoding regulatory variants in cancer. *Genome biology* **15**, 480, doi:10.1186/s13059-014-0480-5 (2014).

16 Smedley, D. *et al.* A Whole-Genome Analysis Framework for Effective Identification of Pathogenic Regulatory Variants in Mendelian Disease. *American journal of human genetics* **99**, 595-606, doi:10.1016/j.ajhg.2016.07.005 (2016).

17 Kircher, M. *et al.* A general framework for estimating the relative pathogenicity of human genetic variants. *Nature genetics* **46**, 310-315, doi:10.1038/ng.2892 (2014).
