# Supplementary material for: RNF43/ZNRF3 loss predisposes to hepatocellular-carcinoma by impairing liver regeneration and altering the liver lipid metabolic ground-state
Source: Nat Commun. 2022 Jan 17;13:334. doi: 10.1038/s41467-021-27923-z (PMC8764073; doi:10.1038/s41467-021-27923-z)
Supplement: Supplementary file 3 — Description of Additional Supplementary Files [file 41467_2021_27923_MOESM3_ESM.docx]

Description of Additional Supplementary Files

Title: Supplementary Data 1.

Description: RNA sequencing data and analysis.

Title: Supplementary Data 2.

Description: IPA analysis of gene expression.

Title: Supplementary Data 3.

Description: Gene expression analysis of lipid metabolism.

Title: Supplementary Data 4.

Description: Wet lipidomics raw data.

Title: Supplementary Data 5.

Description: Comparison of gene expression between different damage models.

Title: Supplementary Data 6.

Description: Human mutation survival and gene expression analysis.

Title: Supplementary Data 7.

Description: List of material
