# Supplementary material for: RNF43/ZNRF3 loss predisposes to hepatocellular-carcinoma by impairing liver regeneration and altering the liver lipid metabolic ground-state
Source: Nat Commun. 2022 Jan 17;13:334. doi: 10.1038/s41467-021-27923-z (PMC8764073; doi:10.1038/s41467-021-27923-z)
Supplement: Supplementary file 11 — Reporting Summary [file 41467_2021_27923_MOESM11_ESM.pdf]

nature research

Corresponding author(s): Meritxell huch

Last updated by author(s): Dec 11, 2021

Reporting Summary

Nature Research wishes to improve the reproducibility of the work that we publish. This form provides structure for consistency and transparency in reporting. For further information on Nature Research policies, see Authors & Referees and the Editorial Policy Checklist .

Statistics

For all statistical analyses, confirm that the following items are present in the figure legend, table legend, main text, or Methods section.

n/a	Confirmed	
☐	☒	The exact sample size (*n*) for each experimental group/condition, given as a discrete number and unit of measurement
☐	☒	A statement on whether measurements were taken from distinct samples or whether the same sample was measured repeatedly
☐	☒	The statistical test(s) used AND whether they are one- or two-sided<br>*Only common tests should be described solely by name; describe more complex techniques in the Methods section.*
☐	☒	A description of all covariates tested
☐	☒	A description of any assumptions or corrections, such as tests of normality and adjustment for multiple comparisons
☐	☒	A full description of the statistical parameters including central tendency (e.g. means) or other basic estimates (e.g. regression coefficient) AND variation (e.g. standard deviation) or associated estimates of uncertainty (e.g. confidence intervals)
☐	☒	For null hypothesis testing, the test statistic (e.g. *F*, *t*, *r*) with confidence intervals, effect sizes, degrees of freedom and *P* value noted<br>*Give P values as exact values whenever suitable.*
☒	☐	For Bayesian analysis, information on the choice of priors and Markov chain Monte Carlo settings
☐	☒	For hierarchical and complex designs, identification of the appropriate level for tests and full reporting of outcomes
☐	☒	Estimates of effect sizes (e.g. Cohen's *d*, Pearson's *r*), indicating how they were calculated

Our web collection on statistics for biologists contains articles on many of the points above.

Software and code

Policy information about availability of computer code

Data collection	Xcalibur software (version 4.1)<br>SONY Sorter SH800S<br>Olympus Fluoview FV1000<br>BioRad CFX Manager<br>LAS V4.13<br>Illumina Hiseq 4000
Data analysis	FlowJo_V10<br>ImageJ v1.51j8<br>SIMCA 14 (Umetrics, Sweden)<br>Metaboanalyst version 4.0<br>Graphpad Prism 8<br>MSiReader v1.0<br>LIPID MAPS® structure database<br>Sickle (version 1.33)<br>STAR aligner (version 2.5.0a)<br>featureCounts (version 1.6.0)<br>ChIPseeker (version 1.12.1)<br>Venny 2.1<br>GSEA 4.0.3<br>biomaRt (version 2.42.0)<br>R 4.1<br>ClustVis

DAVID 6.8
Ingenuity Pathway Analysis (Qiagen)
Leica Application Suite X software (LAS X, v1.5.1.1387)

For manuscripts utilizing custom algorithms or software that are central to the research but not yet described in published literature, software must be made available to editors/reviewers. We strongly encourage code deposition in a community repository (e.g. GitHub). See the Nature Research guidelines for submitting code & software for further information.

Data

Policy information about availability of data

All manuscripts must include a data availability statement. This statement should provide the following information, where applicable:

- Accession codes, unique identifiers, or web links for publicly available datasets
- A list of figures that have associated raw data
- A description of any restrictions on data availability

All RNAseq data are available at the Gene Expression Omnibus (GEO) under accession number GSE133213.
LIPID MAPS® Structure Database https://www.lipidmaps.org/data/structure/
LICA-FR and LIRI-JP human datasets were obtained from ICGC databases: https://daco.icgc.org/
TCF4 targets were determined using datasets from Boj et al. 2012, GSE41284

Field-specific reporting

Please select the one below that is the best fit for your research. If you are not sure, read the appropriate sections before making your selection.

[x] Life sciences [ ] Behavioural & social sciences [ ] Ecological, evolutionary & environmental sciences

For a reference copy of the document with all sections, see nature.com/documents/nr-reporting-summary-flat.pdf

Life sciences study design

All studies must disclose on these points even when the disclosure is negative.

Sample size	We have calculated the sample size using EDA (Experimental Design Assistant tool), as a resource, and redefined on the basis on previous studies performed.
Data exclusions	No data were excluded from the analysis.
Replication	All experiments were replicated at least 2-3 times independently or have at least 2 biological replicates. All attempts at replication were successful
Randomization	When performing animal experiments, animals of the same genotype were randomly allocated to either treated or non-treated groups.
Blinding	Investigators were blinded in all analyses of outcomes of in vivo experiments and organoid lines (e.g. count of proliferating cells in a field) by not knowing genotype of the samples analysed

Reporting for specific materials, systems and methods

We require information from authors about some types of materials, experimental systems and methods used in many studies. Here, indicate whether each material, system or method listed is relevant to your study. If you are not sure if a list item applies to your research, read the appropriate section before selecting a response.

Materials & experimental systems

n/a	Involved in the study	
[ ]	[x]	Antibodies
[x]	[ ]	Eukaryotic cell lines
[x]	[ ]	Palaeontology
[ ]	[x]	Animals and other organisms
[x]	[ ]	Human research participants
[x]	[ ]	Clinical data

Methods

n/a	Involved in the study	
[x]	[ ]	ChIP-seq
[ ]	[x]	Flow cytometry
[x]	[ ]	MRI-based neuroimaging

Antibodies

Antibodies used	Below are reported antibodies used, supplier and product code:<br>GS BD Biosciences 610517<br>Ki67 Thermo Fisher Scientific RM-9106-S1

Pck Dako Z0622
Hmgb1 Abcam ab18256
Cd34 Abcam ab81289
EpCam Thermo Fisher Scientific 14-9326-82
EpCAM-APC eBioscience 17-5791-82
CD31-PE-Cy7 AbCAM ab46733
CD11b-PE-Cy7 BD Bioscience 552850
p21 Abcam EPR18021
Cleaved Caspase-3 Cell Signaling #9664
p-MLKL (Ser345) Abcam ab196436
Cyp2e1 Atlas Antibodies HPA009128
Collagen IV Abcam ab6586
SMA Abcam ab5694
B-Catenin BD 610153
CD3-FITC Biolegend 100203
CD19-BV421 Biolegend 115549
CD64-APC Biolegend 139305
CD49b-PeCy7 Biolegend 103517
Phalloidin-A647 Invitrogen A-22287
Lpcat2 Sigma-Aldrich HPA007891
Albumin SantaCruz sc271605
Anti-Rabbit IgG Alexa Fluor 488 ThermoFischer Scientific A10042
Anti-Rabbit IgG Alexa fluor 568 ThermoFischer Scientific A21447
Anti-Goat IgG Alexa fluor 647 ThermoFischer Scientific A21202
Anti-Mouse IgG Alexa fluor 488 ThermoFischer Scientific A31571
Anti-Mouse IgG Alexa fluor 647 ThermoFischer Scientific H3570

Validation

GS BD Biosciences 610517---validated in Planas Paz 2016
Ki67 Thermo Fisher Scientific RM-9106-S1----validated in Broutier et al 2017
Pck Dako Z0622---validated in Huch et al 2013
Hmgb1 Abcam ab18256---validated in Raven et al 2018
p21 Abcam EPR18021---validated in Raven et al 2018
Cd34 Abcam ab81289---validated in mouse for IHC-P, guarented by company
Lpcat2 Sigma-Aldrich HPA007891---validated in Hall et al 2017
p-MLKL (Ser345) Abcam ab196436---validated in mouse for IF, guarented by company
Cyp2e1 Atlas Antibodies HPA009128---validated in mouse for IHC-P by company as follows: Orthogonal validation of protein expression using IHC by comparison to RNA-seq data of corresponding target in high and low expression tissues and Validation of protein expression in IHC by comparing independent antibodies targeting different epitopes of the protein and Validated against independent antibody Anti-CYP2E1 HPA029564.
Collagen IV Abcam ab6586---validated in mouse for IHC-P, guarented by company
CD3-FITC Biolegend 100203---FC quality tested by company. Each lot of this antibody is quality control tested by immunofluorescent staining with flow cytometric analysis.
CD19-BV421 Biolegend 115549---FC quality tested by company. Each lot of this antibody is quality control tested by immunofluorescent staining with flow cytometric analysis.
CD64-APC Biolegend 139305---FC quality tested by company. Each lot of this antibody is quality control tested by immunofluorescent staining with flow cytometric analysis.
CD49b-PeCy7 Biolegend 103517---FC quality tested by company. Each lot of this antibody is quality control tested by immunofluorescent staining with flow cytometric analysis.
All the below validated in Aloia et al 2019:
EpCAM-APC eBioscience 17-5791-82
CD31-PE-Cy7 AbCAM ab46733
CD11b-PE-Cy7 BD Bioscience 552850
All the below validated in Cordero-Espinosa et al 2021:
SMA Abcam ab5694
B-Catenin BD 610153
Phalloidin-A647 Invitrogen A-22287
Cleaved Caspase-3 Cell Signaling #9664
Albumin SantaCruz sc271605
Anti-Rabbit IgG Alexa Fluor 488 ThermoFischer Scientific A10042
Anti-Rabbit IgG Alexa fluor 568 ThermoFischer Scientific A21447
Anti-Goat IgG Alexa fluor 647 ThermoFischer Scientific A21202
Anti-Mouse IgG Alexa fluor 488 ThermoFischer Scientific A31571
Anti-Mouse IgG Alexa fluor 647 ThermoFischer Scientific H3570

Animals and other organisms

Policy information about studies involving animals; ARRIVE guidelines recommended for reporting animal research

Laboratory animals	All mice, both male and female, involved in the study were Bl6 and aged between 8 weeks and 10 months.
Wild animals	No wild animals were used in the study
Field-collected samples	No field collected samples were used in the study.
Ethics oversight	All mouse experiments were conducted in accordance with procedures approved by the UK Home Office relating to the use of Animals in research (Animals Act 1986).

Note that full information on the approval of the study protocol must also be provided in the manuscript.

Flow Cytometry

Plots

Confirm that:

- [x] The axis labels state the marker and fluorochrome used (e.g. CD4-FITC).
- [x] The axis scales are clearly visible. Include numbers along axes only for bottom left plot of group (a 'group' is an analysis of identical markers).
- [x] All plots are contour plots with outliers or pseudocolor plots.
- [x] A numerical value for number of cells or percentage (with statistics) is provided.

Methodology

Sample preparation	Sample preparation was done accordingly to Broutier et al., Nat Protocols, 2016. Briefly, Healthy livers were isolated and digested with a solution of collagenase and dispase (0.125mg/ml in DMEM/F12) with DNAse. After 3h digestion, biliary ducts became enriched in the preparation and single cells were obtained by incubation with TryplE (Gibco) for 10min and then stained with a pool of surface antibodies conjugated with fluorochromes (see antibody section).
Instrument	SONY SH800S Cell sorter
Software	FowJO_V10
Cell population abundance	Cell population abundance is reported in the Figure as a graph next to the representative plots. CD3+ cells represented around 1% of total viable cells; CD19- representeded less than 0.01% of total viable cells; CD64+ cells and CD3-/49b+ each represented less than 0.5% of total viable cells.
Gating strategy	A population of single cells were sequentially gated based on cell size (forward scatter, FSC, versus side scatter, SSC) and singlets (SSC-Area vs SSC-Height). From these, dead cells were excluded by DAPI+ staining and the resultant population was considered as the negative control for the different markers used: CD3-FITC for T-cells, CD19-BV421 for B-Cells, CD64-APC for macrophages and CD49b-PeCy7 for natural killer cells. CD3-FITC, CD19-BV421 and CD64-APC single fuorescences were compared to PE channel to exclude auto-fluorescence populations and select the positive population while CD49b-PeCy7 fluorescence was compared to CD3-FITC intensity to exclude a sub-population of CD3+/CD49+ T-cells while gating CD3-/CD49+ NK cells.

- [x] Tick this box to confirm that a figure exemplifying the gating strategy is provided in the Supplementary Information.
